# Evidence for two domestication lineages supporting a middle-eastern origin for *Brassica oleracea* crops from diversified kale populations

**DOI:** 10.1093/hr/uhac033

**Published:** 2022-02-19

**Authors:** Chengcheng Cai, Johan Bucher, Freek T Bakker, Guusje Bonnema

**Affiliations:** Plant Breeding, Wageningen University and Research, 6708 PB, Wageningen, The Netherlands; Graduate School Experimental Plant Sciences, Wageningen University and Research, 6708 PB, Wageningen, The Netherlands; Plant Breeding, Wageningen University and Research, 6708 PB, Wageningen, The Netherlands; Biosystematics Group, Wageningen University and Research, 6708 PB, Wageningen, The Netherlands; Plant Breeding, Wageningen University and Research, 6708 PB, Wageningen, The Netherlands

## Abstract

*Brassica oleracea* displays enormous phenotypic variation, including vegetables like cabbage, broccoli, cauliflower, kohlrabi, kales etc. Its domestication has not been clarified, despite several genetic studies and investigations of ancient literature. We used 14 152 high-quality SNP markers for population genetic studies and species-tree estimation (treating morphotypes as “species”) using SVD-quartets coalescent-modelling of a collection of 912 globally distributed accessions representing ten morphotypes of *B. oleracea*, wild *B. oleracea* accessions and nine related C9 Brassica species. Our genealogical tree provided evidence for two domestication lineages, the “leafy head” lineage (LHL) and the “arrested inflorescence” lineage (AIL). It also showed that kales are polyphyletic with regards to *B. oleracea* morphotypes, which fits ancient literature describing highly diverse kale types at around 400 BC. The SVD-quartets species tree topology showed that different kale clades are sister to either the LHL or the AIL. Cabbages from the middle-east formed the first-branching cabbage-clade, supporting the hypothesis that cabbage domestication started in the middle-east, which is confirmed by archeological evidence and historic writings. We hypothesize that cabbages and cauliflowers stem from kales introduced from Western Europe to the middle-east, possibly transported with the tin-trade routes in the Bronze age, to be re-introduced later into Europe. Cauliflower is the least diverse morphotype showing strong genetic differentiation with other morphotypes except broccoli, suggesting a strong genetic bottleneck. Genetic diversity reduced from landraces to modern hybrids for almost all morphotypes. This comprehensive Brassica C-group germplasm collection provides valuable genetic resources and a sound basis for *B. oleracea* breeding.

## Introduction

*B. oleracea* is an important vegetable and fodder crop species and exhibits enormous diversity in its appearances, including the leafy heading morphotypes var. *capitata* (cabbage), the typical curd subspecies with large arrested inflorescences, including var. *botrytis* (cauliflower) and var. *italica* (broccoli), the kohlrabi’s with their tuberous stems (var. *gongylodes*), the Brussels sprouts with their axillary buds (var. *gemmifera*), and other leafy vegetable types with different shapes, such as var. *viridis* (Collard green), var. *alboglabra* (Chinese kale) [1, 2], var. *costata* (Tronchuda kale) [3] as well as other types of kale, such as *var. acephala* (bore and curly kale, marrow stem kale, etc). In addition, var. *acephela* (ornamental kale) has been bred by intercrossing different morphotypes [4]. Different *B. oleracea* morphotypes are extensively cultivated, with several crops cultivated almost worldwide (like cabbages and cauliflowers), while others in specific countries only depending on local preferences (like Tronchuda’s in Portugal and Collards in the USA). Despite this enormous diversity, *B. oleracea* is still considered as one species and morphotypes can be easily interbred.

Wild *B. oleracea* can be found along the Atlantic and Mediterranean coasts, all the way from Norway to England and France down to Greece. Wild *B. oleracea* plants form a strong stem with large waxy leaves, and only flower after several winters [1, 2, 5]. *B. oleracea* and its closely-related wild relatives belong to the so-called “C-genome group”, which is characterized by the possession of nine chromosome pairs [6]. These wild “C9 species”, which probably form a monophyletic group, mainly occur in the Mediterranean region, particularly in Sicily [7, 8]. In his doctoral thesis, Maggioni summarized the above-mentioned origin and morphological characteristics of these wild species [6]. In short, they are characterized by hairy leaves, petiolated or with wings, and/or yellow flowers, such as in *Brassica incana*, *Brassica villosa*, *Brassica macrocarpa* and *Brassica rupestris*. Phenotypic diversity of wild relatives of *B. oleracea* has been assessed within the European Cooperative Program for Plant genetic Resources (ECPGR). A few hybridization experiments have been performed to investigate relationships between wild “C9 species” and cultivated forms of *B. oleracea* [9,10]. Kianian and Quiros (1992) [9] analysed fertility and meiotic chromosome behaviour from 172 intra- and interspecific hybrids of *B. oleracea* crops and wild “C9 species”. Based on their experiments, fertile hybrids can be produced through crosses of *Brassica alboglabra*, *Brassica bourgeaui, Brassica cretica, Brassica montana* to *B. oleracea* crops. Crosses of *Brassica incana, Brassica insularis and Brassica rupestris* to *B. oleracea* crops produced semi-sterile progenies, which was associated with abnormal meiotic behaviour. Crossing experiments from von Bothmer et al. (1995) [10] also indicated possibly frequent introgression between wild “C9 species” and *B. oleracea* crops. In a recent study, Mabry et al. (2021) investigated the evolutionary history of wild, feral and domesticated *B. oleracea*, including nine C9 species which they refer to as “progenitor species”. The authors identify the Aegean endemic *B. cretica* as closest living relative of *B. oleracea* [11].

Domestication of *B. oleracea* dates back to as early as around 400 BC, based on for example descriptions of kales by the Greek scholar Theophrastrus (370–285 BC). In older writings (800–600 BC) the kales were not yet mentioned [12, 13]. Northwestern Europe and the Mediterranean/Middle east regions are hypothetical ancestral areas for domesticated *B. oleracea*, of which the latter obtains more weight from ancient literature [2, 13]. Also Mabry et al. (2021) see the identification of the Aegean endemic *B. cretica* as closest living relative of *B. oleracea* as support for a Eastern Mediterranean domestication origin for *B. oleracea*. Gomez-Campo and Prakash [14], suggest several possible domestication routes, one of them being that kales were the earliest cultivated forms of *B. oleracea*, cultivated along the Atlantic and Mediterranean coasts by the Celts, from where they were brought to the East Mediterranean region (first and second millenia BC) to become domesticated. John Gerard (1597) describes in his herbal that Theophrastus, and the Romans Cato (234–149 BC) and Pliny the Elder (23–79 AD) already described “wild and tame coles”, and distinguished several “coleworts” (kales). These included the smooth, great, broad-leaved, with a big stalk type, the ruffed type and types with little stalks that are tender and “very much biting”. One hypothesis is that these diverse kales (“coleworts”) were transported along tin trade routes (Bronze Age, around 3300–1000 BC) from Spain and the British islands to the Phoenicians (present-day Libanon), who referred to *B. oleracea* crops as “Krambe” [15, 16]. The name Krambe was later used by Linnaeus for the genus *Crambe*, which is closely-related to Brassica [17], and is known for its coastal distribution and fleshy cabbage-like leaves. If indeed kales were introduced to the middle-east, it is also possible that domestication of cabbages and cauliflowers initiated there. In addition to kales, cabbages (“coles”) are also first mentioned around the first century AD. In Maggioni (2018) we can read that Pliny the Elder (23–79 AD) already describes coles that “grow so big that a poor man’s table would not be large enough to hold it”. Lucius Junius Moderatus Columella (4–ca. 70 AD), born in Spain, but a military officer in the Roman Legion and later settled as landowner in Italy, described many different “caules” based on where they were grown. He also mentions the sprouting types (cymae), which were mentioned by Pliny the Elder too. These refer to axillary buds that are tender, and indicate that apical dominance was less pronounced as it is in modern cultivars. Around the same times, cauliflowers are described, as *Brassica cypria* in Latin, and as “cauliflore” in Italian, which seems to agree with *Brassica pompeiana* described by Pliny the Elder [13]. The Spanish Arabian author Ibn-Al-Awan (c.1140), was the first to distinguish heading and sprouting cauliflowers. He named cauliflower “quarnabit”, the present Arabic name for cauliflower, suggesting Syria as center of origin, while the herbalist Dodonaeus (1578) suggested Cyprus. Crisp (1982) hypothesized that cauliflowers are evolved from broccoli’s based on crossing experiments. In a recent study by Guo et al. (2021), this was also suggested based on their inflorescence phenotypes and causal mutations [18]. Dale-Champ (1586) writes that cauliflowers have evolved between 400–600 BC and are believed to have diversified in the Eastern Mediterranean, and from there introduced to Italy [14]. Maggioni et al (2018) however did not find any mentioning of “cauliflower” till the first century. Dodonaeus (1578) and also John Gerard (1597) describe and illustrate in detailed drawings of white, red and savoy cabbages, cauliflower and kale but kohlrabi is not mentioned [19].

To date, several genetic diversity and population structure studies have been performed and published for domesticated *B. oleracea* [11,20–24]. Several studies are limited by low numbers of markers [20–22] and, more importantly, hardly any studies include all described *B. oleracea* morphotypes, and the ones that are included are often represented by low numbers of accessions [23, 25, 26]. What generally lacks in these studies is data on genetic comparisons between modern hybrid and old landrace accessions. Recently, Cheng et al. resequenced 119 *B. oleracea* and 199 *Brassica rapa* accessions representing seven resp. 11 morphotypes, to study their genetic diversity and genealogical relationships [24]. They showed that the cauliflower and broccoli accessions formed a separate cluster, with a long branch length separating it from a cluster comprising cabbage, ornamental kale, Brussels sprouts and kohlrabi accessions. Only few published studies also include *B. oleracea* wild C9 relatives which can be intercrossed with domesticated *B. oleracea* crops [11, 27].

**Table 1:** Summary of all the accessions used in this study.

**Morphotype**	**genebank**	**hybrids**	**others**	**Total**
**Broccoli**	**47**	**52**	**1**	**100**
*summer-autumn*	*16*	*35*	*0*	51
*unkown*	*16*	*1*	*1*	18
*winter*	*15*	*16*	*0*	31
**Cauliflower**	**84**	**138**	**1**	**223**
*romanesco*	*5*	*4*	*0*	9
*summer-autumn*	*28*	*84*	*0*	112
*tropical*	*9*	*13*	*0*	22
*unkown*	*24*	*2*	*1*	27
*winter*	*18*	*35*	*0*	53
Collard Green	20	0	0	20
**Heading cabbage**	**180**	**130**	**1**	**311**
*pointed*	*4*	*6*	*0*	10
*red*	*23*	*21*	*0*	44
*savoy*	*40*	*12*	*0*	52
*unkown*	*5*	*5*	*0*	10
*white*	*108*	*86*	*1*	195
Chinese Kale	10	1	0	11
**Kale**	**29**	**5**	**0**	**34**
*kale*	*8*	*0*	*0*	8
*bore-curly*	*11*	*4*	*0*	15
*marrow-stem*	*10*	*1*	*0*	11
Kohlrabi	34	17	1	52
Ornamental	2	24	0	26
Sprouts	39	10	0	49
Tronchuda	33	0	0	33
Wild oleracea	20	0	0	20
Wild C9	33	0	0	33
**Total**	**531**	**377**	**4**	**912**

Our aim was to study genealogical relationships, population structure and domestication history of *B. oleracea* morphotypes, based on dense lineage- and character-sampling. For this purpose we generated a collection of 912 accessions representing the majority of morphotypes of *B. oleracea*, including both modern hybrids and old landraces with worldwide geographical origins, and its wild relatives (C9 species). Using genotypic data (14 152 SNPs), we estimated nucleotide diversity in each group and compared the differentiation between groups. We compared genetic diversity between genebank accessions and modern hybrids as well as between different subgroups such as ecotypes within morphotypes. We estimated species tree topology using coalescent-based population genetics modelling, treating morphotypes as “species”. We provide evidence for two main lineages within the cultivated *B. oleracea*’s, the “leafy head lineage” (LHL; cabbages, collards and ornamentals) and the “arrested inflorescence lineage” (AIL; cauliflower and broccoli). We show that most cauliflower accessions form a monophyletic group, in a position, probably most-derived of all morphotypes and hypothesize that cauliflower domestication went through a strong bottleneck.

## Results

### Global geographic distribution of brassica germplasm

A total of 912 accessions, representing 10 *B. oleracea* morphotypes, wild *B. oleracea* and nine wild C9 species, were selected for genetic diversity analysis (Table 1, Table S1). This germplasm set consists of 377 modern hybrid accessions and 531 accessions with a global geographic representation. As shown in Table S1, germplasm is selected from ~53 countries with the majority from Europe. Broccoli and cauliflower materials are mainly obtained from Italy, the UK and the Netherlands. The collection of heading cabbage has various geographical origins, such as the Netherlands, Germany, the UK, Macedonia, Russia as well as other countries, like Turkey, reflecting the fact that heading cabbages are adapted to a wide range of climatic zones. The Collard green collection includes materials from the USA and Middle-East countries (Turkey and Syria). Most of Brussels sprouts accessions are obtained from the Netherlands, Denmark, Germany and France and the majority of Tronchuda accessions are collected from Portugal and Spain. Wild *B. oleracea* accessions are mainly collected from the UK and wild C9 species from Italy.

### Pairwise genetic distance matrix reveals lower genetic distance within morphotypes and higher genetic variations in genebank accessions compared to hybrids

**Figure 1:**
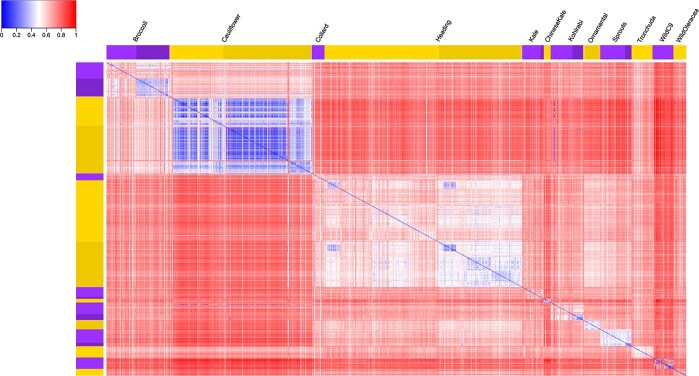
Heatmap showing genetic distance matrix between 912 accessions. For each group, light color indicates genebank accessions, and dark color indicates modern hybrid accessions.

Overall, pairwise genetic distances between morphotypes were larger than within morphotypes (Fig. 1). Interestingly, the lowest pairwise genetic distance within morphotypes was observed in the cauliflower group, illustrating their very low genetic diversity. In addition, the genetic variation in modern hybrid accessions was much lower than that of *B. oleracea* genebank accessions for most morphotypes. However, this was not obvious for cauliflower, with overall low diversity (average normalized genetic distance of 0.37) in both genebank and hybrid accessions.

Based on pairwise genetic distance, we also compared genetic variation between the different ecotypes and cultivar-groups (hereafter referred to as “varieties”) in broccoli, cauliflower, heading cabbage, kale and between the different wild C9 species. Different broccoli ecotypes (summer/autumn, winter and unknown) showed similar levels of genetic variation, therefore the genetic variation between genebank and modern hybrids is larger than that between the different ecotypes (Fig. S2a). However, the cauliflower group exhibited different patterns as differences in genetic variation between ecotypes of cauliflower is greater than between genebank and modern hybrids. Romanesco cauliflower displays ample genetic variation. Variation among winter cauliflower accessions was larger than summer/autumn and tropical types (Fig. S2b). For heading cabbage we distinguish four varieties (red, white, savoy and pointed). Red cabbage is the least diverse subgroup, with little variation in both genebank- and hybrid accessions (Fig. S2c). We observed that there are two subgroups within both the white cabbage genebank and modern hybrid accessions, which showed genetic differentiation. We further investigated pairwise genetic distance within the wild C9 species group. A few accessions behaved unexpectedly as they differed extensively from their peer accessions. This might be due to incorrect classification of genebank materials (Fig. S2e, Supplementary Notes) .

### Genome-wide diversity comparisons among groups shows that cauliflower is the least diverse morphotype

**Figure 2:**
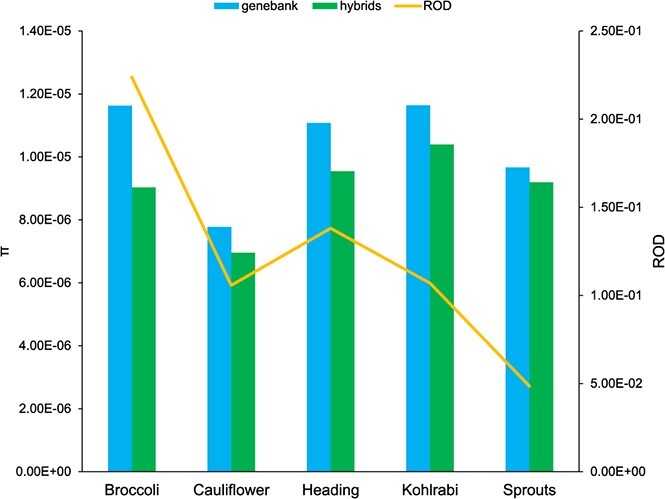
Comparison of nucleotide diversity (π) and reduction of diversity (ROD) between genebank and modern hybrid accessions in five groups. The genebank group was used as the control for ROD calculation in this figure.

We compared genome-wide nucleotide diversity (π), reduction of diversity (ROD) and pairwise population differentiation level (F_ST_) between and within morphotype groups (Table S4). Among all the morphotypes, cauliflower had the lowest mean nucleotide diversity (π_cau_ = 7.13 × 10^−6^), which is 46% lower than the highest value identified in wild *B. oleracea* (π_wbo_ = 1.32 × 10^−5^). The nucleotide diversity of kale was slightly lower (6%) than the figure of wild *B. oleracea*. Π decreased from genebank accessions to hybrid accessions in broccoli, cauliflower, heading cabbage, kohlrabi and Brussels sprouts, with the highest ROD detected in broccoli (ROD_bro_ = 2.24 × 10^−1^). This is consistent with the finding of pairwise genetic distance even though for cauliflower the differentiation was not obviously shown in the heatmap (Fig. 1 and 2). F_ST_ values between cauliflower and other morphotype groups ranged from strong (0.21 for heading cabbage) to very strong (0.34 for wild C9 species), with the exception of broccoli (0.15; moderate differentiation) (Table 2). Notably, wild *B. oleracea* had very strong differentiation with cauliflower (0.31), strong differentiation with Chinese kale (0.17) while moderate differentiation with all other *B. oleracea* morphotypes. Based on this we conclude that genetic differentiation between cauliflower and the other morphotypes including wild *B. oleracea* was larger than between wild *B. oleracea* and the rest. Wild C9 species had moderate differentiation from wild *B. oleracea*, but had strong or very strong differentiation with other *B. oleracea* morphotypes. F_ST_ analyses revealed little genetic differentiation between summer/autumn and winter broccoli (F_ST_ = 0.04) (Table S5). Cauliflower ecotypes showed little and moderate differentiation with each other (F_ST_: 0.02 ~ 0.07). Red cabbages had moderate differentiation with all other heading cabbage varieties (F_ST_: 0.06 ~ 0.09) whereas only little differentiation was present among pointed, savoy and white cabbages (0.03 ~ 0.04).

**Table 2:** Pairwise comparison of F_ST_ values between different morphotype groups

**Group**	**Broccoli**	**Cauliflower**	**Collard**	**Heading**	**Kale**	**KaleChinese**	**Kohlrabi**	**Ornamental**	**Sprouts**	**Tronchuda**	**WildC9**	**WildOleracea**
**Broccoli**	-	0.15	0.13	0.15	0.12	0.20	0.12	0.16	0.18	0.10	0.22	0.14
**Cauliflower**	-	-	0.28	0.21	0.28	0.28	0.24	0.29	0.28	0.23	0.34	0.31
**Collard**	-	-	-	0.05	0.04	0.17	0.08	0.09	0.13	0.07	0.18	0.04
**Heading**	-	-	-	-	0.10	0.19	0.11	0.08	0.11	0.12	0.22	0.11
**Kale**	-	-	-	-	-	0.18	0.08	0.10	0.12	0.06	0.15	0.02
**KaleChinese**	-	-	-	-	-	-	0.19	0.26	0.27	0.15	0.29	0.17
**Kohlrabi**	-	-	-	-	-	-	-	0.13	0.15	0.10	0.20	0.09
**Ornamental**	-	-	-	-	-	-	-	-	0.16	0.13	0.23	0.11
**Sprouts**	-	-	-	-	-	-	-	-	-	0.15	0.23	0.13
**Tronchuda**	-	-	-	-	-	-	-	-	-	-	0.19	0.07
**WildC9**	-	-	-	-	-	-	-	-	-	-	-	0.15
**WildOleracea**	-	-	-	-	-	-	-	-	-	-	-	-

**Figure 3:**
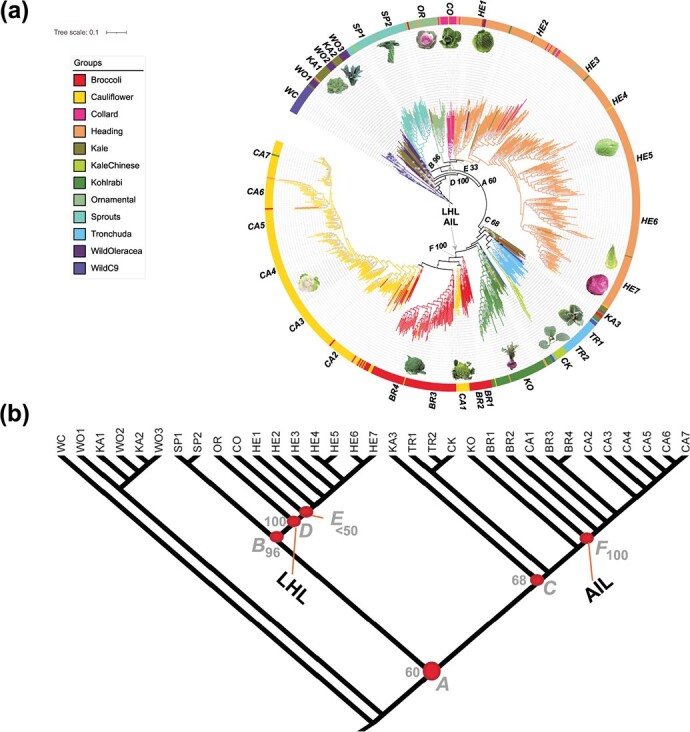
Genealogy of major *Brassica oleracea* morphotypes and wild C9 species. (a) Maximum likelihood tree of *B. oleracea* and wild C9 species accessions inferred from 14 152 high-quality SNPs. Grey arrows indicate the two main cultivated lineages, AIL and LHL, respectively. Labels A-F show selected deep nodes with numbers representing bootstrap values. Labels outside circle refer to clade names (the abbreviations: WC: wild C9 species, WO1-WO3: wild *B. oleracea*, KA1-KA3: kale, SP1-SP2: Brussels sprouts, OR: ornamental kale, CO: collard, HE1-HE7: heading cabbage, TR1-TR2: tronchuda, CK: Chinese kale, KO: kohlrabi, BR1-BR4: broccoli, CA1-CA7: cauliflower). The circled blue dots in clades represent bootstrap values of above 80%. Pictures of morphotypes were obtained from Cheng et al. [28] and Google. (b) A schematic tree depicting the ML tree structure.

### Genealogical relationships reveal two main lineages for cultivated *B. oleracea* morphotypes

We constructed a *B. oleracea* genealogy tree including 912 accessions (879 *B. oleracea* and 33 wild C9 species), using IQ-TREE maximum likelihood analysis which selected the GTR + F + ASC + R10 model as best-fitting based on BIC. The monophyletic group including the vast majority of wild C9 species samples was set as outgroup. Our analysis revealed that the wild *B. oleracea* and kales (WO1–3 and KA1–2) together formed a paraphyletic group, and the first node following (node A; bootstrap support 60%) represents the start of a “cultivated *B. oleracea* lineage” (named here) (Fig. 3). At node A all the cultivated morphotypes are divided over two main lineages. In one direction this includes several leafy types, that subtend at node B (bs 96%) in the Brussels Sprouts and the “leafy head lineage” (*LHL*), which includes besides cabbages, collards and ornamentals. The other direction includes few kales (KA3), tronchuda’s, Chinese kale and kohlrabi, and the “arrested inflorescence lineage” (*AIL*, F, bs 100%) which includes cauliflowers and broccoli’s. At node B (bs 96%) the tree subtends into the monophyletic sprouts (SP1 and SP2; bs 100%) and the LHL at node D (bs 100%). Node D subtends the ornamentals (OR, bs 100%) and at node E (bs 33%) the remaining “leafy” clades (CO, HE1-HE7; HE = heading). The Collards are sister to the cabbage clade but lacking support; the cabbage clade splits into HE1 and HE2–7 (bs 80%), and individual clades HE2-H7 are well supported. HE1 is mainly composed of savoy cabbages, HE2-HE6 of white cabbages and HE7 of red and pointed cabbages. Interestingly, HE2 is composed of germplasm accessions from Eastern Mediterranean countries, as do the few Collards that are included in HE2. In the other main lineage node F (bs 100%) demarcates the split between the cauliflowers and broccoli’s on the one hand, and the kohlrabi, tronchuda’s, Chinese kale and few kales on the other hand. In this very diverse group, the first branchpoint is towards several kales (no support), followed by a branchpoint towards two tronchuda clades (TR1 without support and TR2 (bs 100%)), with nested in it a small Chinese kale clade (CK; bs 100%). The kohlrabi’s form a monophyletic group. In the cauliflower-broccoli clades, several broccoli clades (BR1–4) were interspersed with a Romanesco clade (CA1), but support for this placement is low (bs 59%). From here we see a branch towards the most derived clades, the cauliflowers, with CA2 mainly representing winter cauliflowers, CA3 a mixture of winter and summer/autumn cauliflowers, CA4 and CA5 the summer/autumn types, and CA6 and CA7 the tropical and summer/autumn cauliflowers.

**Figure 4:**
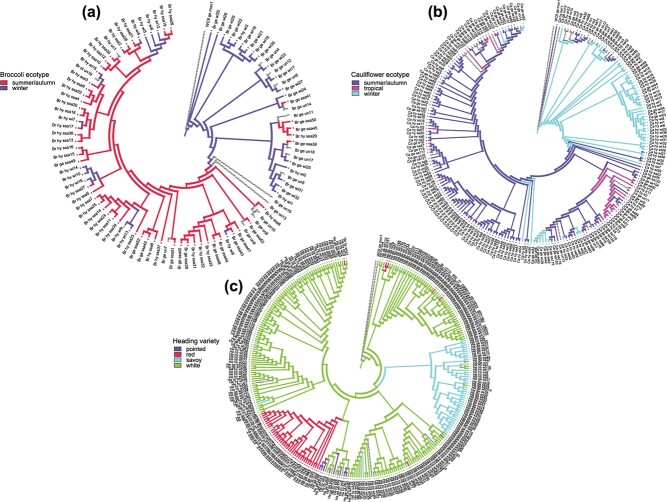
Ecotype/variety character evolution for (a) broccoli, (b) cauliflower and (c) heading cabbage. Different varieties of heading cabbage were treated as the states of variety character, and different ecotypes of broccoli or cauliflower were treated as the states of ecotype character. Grey branches represent accessions with “unknown” state.

### Ancestral character state reconstruction shows that in cabbages the white cabbage variety is ancestral to red, savoy and pointed, while in broccoli and cauliflower the winter ecotypes are ancestral

Ancestral character state reconstruction for heading cabbage suggests that white cabbage is the ancestral state. Both savoy and red cabbage are derived from white cabbage (Fig. 4c). Winter broccoli is suggested as the ancestral state, giving rise to summer/autumn broccoli. It appears that broccoli experiences reverse evolution from summer/autumn to winter broccoli which might be caused by breeding activities (Fig. 4a). The ancestral character state reconstruction also implies winter cauliflower as the ancestral state, with summer/autumn cauliflower derived from winter cauliflower. There is a transition from summer/autumn cauliflower to tropical cauliflower, and also in cauliflower we see this “reverse evolution” as tropical cauliflower then gives rise to summer/autumn cauliflower, followed by winter cauliflower (Fig. 4b). In general, we conclude that the predominant trend for ecotype character is towards summer/autumn from winter and that reversals are rare. We also analysed ancestral state reconstruction with states defined as the different geographical origins. For hybrids this information is difficult to interpret, as hybrids origin leads to breeding company and not necessary to growing area. For the cauliflowers, accessions from Southwest Europe (mainly from Italy, with many romanesco’s) formed the ancestral state (Fig. S3b). For broccoli’s, the ancestral state is formed by accessions from Northwest Europe, with mainly winter broccoli accessions from Great Britain (Fig. S3a). For the heading cabbages, accessions from Southeast Europe, mainly Macedonia (MKD) and Yugoslavia (YUG) formed the ancestral state (Fig. S3c), pointing to an east Mediterranean/near-east origin.

### Principal component analysis and population structure support genealogical relationships revealed by ML tree

**Figure 5:**
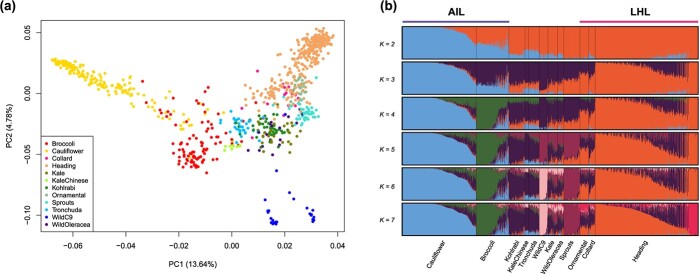
PCA and population structure. (a) PCA plots of 875 accessions using whole genome SNP data. The first two principle components were plotted to visualize the relationships among individuals (accessions) and groups. (b) Population structure of major *Brassica oleracea* morphotypes and wild C9 species with different numbers of clusters (*K = 2–7*). Each accession is represented by a vertical bar. The different colors represent contributions to the K-groups. The length of each colored segment in the bar quantifies cluster membership.

The genealogical relationships between different groups were also supported by principle component analysis (PCA), with the first two principle components explaining 13.64% and 4.78% total genetic variance, respectively (Fig. 5a). In PC1 AIL with cauliflower and broccoli accessions, is clearly separated from the Brussels sprouts and the LHL (heading cabbage, ornamental and collard accessions). Chinese kale, kohlrabi and Tronchuda accessions, but also the kale accessions are located between these two groups. PC1 also separated wild C9 species into two clusters, which corresponds to the maximum likelihood tree in Fig. S4. PC2 clearly separated wild C9 species from *B. oleracea* wild type and morphotypes. The PCA analysis appeared to corroborate the genealogical tree and population differentiation analyses. Further detailed PCA analysis within morphotype was also in line with genealogical and pairwise genetic distance analyses (Supplementary Notes).

To further investigate population structure of the 875 *B. oleracea* and wild C9 accessions, the STRUCTURE v2.3.4 [29] software was utilized to infer the structure of these accessions. We ran the pipeline by gradually increasing the number of clusters (*K*). The*Δk* analysis revealed that *K = 4* fits the dataset best (Fig. S5). Interestingly, when *K = 2*, cauliflower and not wild *B. oleracea* and wild C9 species, formed one cluster that was clearly separated from other *B. oleracea* morphotypes and these wild C9 species (Fig. 5b). All of the broccoli accessions were admixed and received genetic contributions from cauliflower and other morphotypes. When *K* increased from 2 to 7, kale and wild *B. oleracea*, ornamental kale and Collard green on the one hand and kohlrabi, Chinese kale and tronchuda on the other hand always had similar membership and thus were clustered together, respectively. At *K = 7*, heading cabbage cluster was subdivided into two clusters, with the new cluster formed by 37 red cabbage accessions. Kale and wild *B. oleracea* could not be assigned to independent clusters when *K* ranged from 8 to 12 (Fig. S6). This fits the hypothesis that wild *B. oleracea* and kale are progenitors of different morphotypes, which is also clearly shown from the ML tree (Fig. 3a). Overall, the majority of morphotypes formed a distinct group with gradual increase of the number of clusters. However, more and more accessions within each group become highly admixed, indicating both common ancestry and gene introgression among different groups.

### Species tree and divergence time estimation suggest ancient divergent kale lineages leading to the AIL and LHL lineages

**Figure 6:**
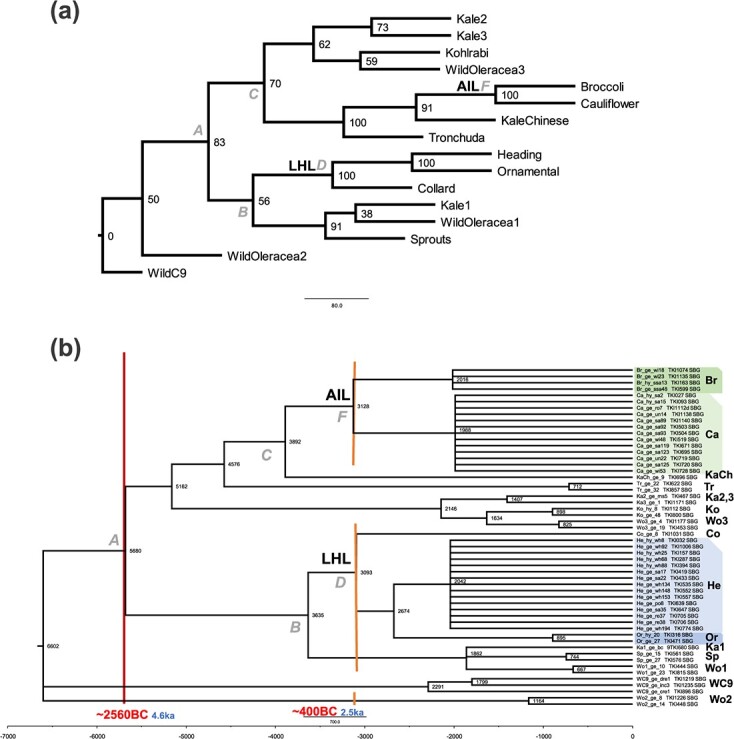
*Brassica oleracea* species tree. (a) SVDquartet analysis of the full 875 terminal SNP matrix; bootstrap values are indicated at the nodes. Grey letters at nodes refer to main clades as distinguished in Fig. 3a – the ML Overall tree. (b) Tree topology of the SVDquartets analysis from (a) with nodes with bootstrap values <50% collapsed, enforced on SUB matrix, and arbitrarily ultrametricized in Mesquite (see text). Numbers at nodes and scalebar indicate relative ages. *B. oleracea* morphotypes are indicated at terminal labels on the right; proposed dating of domestication events based on earliest reports, as well as the timing of tin trades/Bronze Age (see text) are indicated. Grey letters at nodes refer to main clades as distinguished in Fig. 3a–the ML Overall tree. The abbreviations on the right: Br: broccoli, Ca: cauliflower, KaCh: Chinese kale, Tr: tronchuda, Ka1-Ka3: kale, Ko: kohlrabi, Wo1-Wo3: wild *B. oleracea*, Co: collard, He: heading cabbage, Or: ornamental kale, Sp: Brussels sprouts, WC9: wild C9 species.

The SVD-quartets analysis on the “Overall” SNP matrix yielded a tree topology that was not fully congruent with the ML tree. Although the two main lineages AIL and LHL are present (both bs 100%), sprouts are sister to Wildoleracea1 (WO1) and Kale1 (KA1) (bs 91%) and Kale2 and Kale3 (KA2 and KA3) (bs 73%) are sisters in the SVDq topology (Fig. 6a), while in the ML tree WO1 together with WO2 and WO3, and KA1 together with KA2 precede the complete “cultivated *B. oleracea* lineage”. The position of kohlrabi (KO) is problematic as it is sister to Wildoleracea3 which contradicts its position in the overall ML genealogy.

Given the accuracy of SVD-quartets analysis [30, 31] and its use of coalescent modelling (allowing gene trees to be different) (see Supplementary Notes), we consider it to be more accurate than the ML tree topology. We therefore use the SVDq topology to estimate branch lengths on, in order to enable time estimation of nodes. After ultrametricizing the SVDq-based tree with SNP nucleotide branch lengths (Fig. 6b), we see that AIL (node F) would appear of a similar age as LHL (node D). Tronchuda, Kohlrabi and Chinese kale, but also Sprouts, would appear as younger. The main divergence among Cauliflowers in AIL and among Cabbages in LHL appears “simultaneously”. Since our approach to dating the nodes is fairly crude (it does not include molecular clock modelling, rate smoothing nor standard deviations) we interpret our relative node-ages as rough estimates and an indication of relative ancestry of lineages. Nevertheless, we did consult the literature on ancient writings for possible timing of first occurrences of, for instance, cauliflower and cabbages, which were mentioned by Theophrastus and Pliny the Elder at around 400 BC (or 2.5 ka; see Introduction). Applying this date to nodes F and D (which mark the onset of cauliflower/broccoli and heading cabbage diversification breeding, respectively), and assuming clock-like accumulation of SNP’s, we find a SNP-rate of 3100 node height /2500 yr = 1.24 SNP yr^−1^. Applying this to node A (the onset of cultivation of *B. oleracea*) we find an age of 5680 node height/1.24 = 4581 yr, which is 2560 BC. As diversification within the cauliflower and heading cabbage clades is based on accessions of modern hybrids and old landraces with worldwide geographic origins, we feel the above scenario is accurate/realistic to base our time estimates on.

## Discussion

The *Brassica* C-group germplasm collection of 972 samples we generated and genotyped in this paper is the most comprehensive published so far. We included most described *B. oleracea* subspecies, herein referred to as morphotypes and wild *B. oleracea*, but also their *Brassica* C9 relatives. This allows us to 1) perform extensive genetic diversity analysis within and between morphotypes, 2) investigate “tokogenetic” relationships between all *B. oleracea* morphotypes, their wild relatives, as well as different wild C9 species, and 3) infer population structure of this large population. As both genebank and modern hybrid accessions are included we can compare their genetic diversity which is important for breeders to evaluate their potential use for mining novel variation.

*B. oleracea* accessions are generally self-incompatible and for that reason heterogeneous. As we aimed to include a large number of these heterogeneous genebank accessions (531 in our study), we decided to represent accessions by single plants, similar to other studies [11, 32]. A pilot to test how this influences results showed that intra-accession variation is generally smaller than inter-accession variation, but also showed that genotypes/plants from genebank accessions that display inter-plant phenotypic variation are not always differentiated from genetically closely related accessions.

We performed several comparative analyses, and overall the results were congruent. This was especially the case for the ML tree, the PCA and the STRUCTURE analysis. These analyses showed that the cauliflowers had highest genetic differentiation from other morphotypes and the C9 species, and that the broccoli’s were closest to cauliflower. In PCA analyses, cauliflowers separated from all other morphotypes in PC1 (explaining 13.64% of the variation), while C9 species only separated in PC2 (4.78% variation explained). Also in STRUCTURE analysis, at K = 2, one group consisted of cauliflower accessions, and the other group included all remaining accessions. Only at K = 6, the C9 species formed a separate group. Analysis of population differentiation between morphotypes again showed that F_ST_ values between cauliflower and other morphotype groups was higher than between wild *B. oleracea* and other morphotypes. Results of pairwise genetic distance analyses and nucleotide diversity analyses also agreed. Genetic variation among cauliflower accessions was very low. Genetic variation within morphotypes was substantially lower between hybrids than between genebank accessions. This was to a lesser extend the case for cauliflowers. The strong genetic differentiation of cauliflower combined with a reduced genetic variation among cauliflower accessions points to a very strong genetic bottleneck. In a recent publication by Guo et al. (2021) a *de novo* genome assembly of a cauliflower and a cabbage was generated and structural variations shared among cauliflower resp cabbage accessions were exploited to identify possible selection signals. This revealed many “cauliflower” selection signals in diverse molecular pathways (flowering time, floral identity, meristem proliferation, organ size and spirality), suggesting indeed a strong genetic bottleneck.

The SVD-quartets analysis yielded a tree topology that was not fully congruent with the ML tree, which is not unexpected given the coalescent-based nature of the SVDq approach. Both trees revealed two clearly separated cultivated lineages, the LHL and AIL. In any case, we use the SVDq topology in our further discussions given the accuracy of SVD-quartets analysis [30, 31]. This tree shows that the kales form different clades that may represent progenitors of the LHL and AIL. Our separate kale lineages (KA1 vs KA2,3) that diverged before the emergence of LHL and AIL, are consistent with a scenario involving “ancient” divergent kale lineages leading to LHL and AIL. This illustrates that the kales were already diversified at the time when *B. oleracea* domestication started and that they are likely to be ancestral to the other cultivated morphotypes. For possible timing of these domestications, we did consult the literature on ancient writings, to search for first occurrences [13]. For instance, cauliflower and cabbages were mentioned by Theophrastus and Pliny the Elder at around 400 BC (or 2.5 ka; see Introduction); also ancient literature mentions wild and tame “coles”, and distinguished several “coleworts” (kales) based on leaf, stalk type, sprouts and taste. Applying 400 BC to nodes F (AIL) and D (LHL), we find an age of 4581 yr (2560 BC) for node A (i.e. the onset of cultivation of *B. oleracea*). As wild *B. oleracea* grows along the coasts of England, France and Spain, and both our evidence of cabbages (Mesquite analysis based on origin, Fig. S3), and several publications (see Introduction) points to domestication in the middle east, one possible explanation is that ships transporting tin ores from Cornwall and the Iberian island, also carried “coleworts” along. The 2560 BC date for the origin of *B. oleraceae* breeding would be consistent with a time frame of emerging tin-trade between Britain, France, Spain and Portugal to the Mediterranean [15]. Berger et al. (2019) proof that tin ingots excavated around the eastern Mediterranean sea are from Cornish tin mines, based on radiogenic composition that can be linked to geological age of the tin ores. They state that this trade shifted from the Near East to Europe and Cornwall in particular, at the demise of the Minoans and during the rise of the Mycenaeans ca. 1430 BCE, which is slightly later than our estimated time of 2560 BC.

Ancestral state analysis using Mesquite was performed for the cauliflowers, cabbages and broccoli’s separately to understand their breeding history. Winter broccoli and cauliflower types were ancestral to the earlier flowering types, while for cabbages, the white cabbages were inferred to be ancestral. We also reconstructed ancestral areas and this revealed that for cauliflowers, the ancestral types came from Italy, with several Romanesco types at their origin, and for heading cabbages, ancestral types originated from eastern Europe, with many from Macedonia (MKD) and Yugoslavia (YUG), supporting an east Mediterranean/near east origin. For the broccoli’s results were different, as the ancestral types were winter broccoli’s from Great Britain. This fact conflicts with the genealogic tree and previous studies that suggest that cauliflower was derived from broccoli. We included all broccoli accessions from the involved genebanks so cannot expand the data to include accessions of different origins.

SVD-quartets topology also places the Brussels sprouts, sister to kale1 and wild oleracea1, as a separate lineage that emerged later than the cabbages. Former studies also indicated different ancestors for sprouts and cabbages, and often also mentioned wild C9 species in their ancestries (Mabry et al. 2021 and references there in (Schulz 1936; Neufchatel 1927; Helm 1963)). The position of sprouts sister to kales fits their botanical characteristics, with a long stalk and absence of apical dominance (“sprouting coleworths” were mentioned by Cato and Pliny the Elder at around 200 BC (see Introduction)). Kohlrabi’s form a separate lineage sister to kale 2 and 3 clades and wild oleracea 3. Little is known about their domestication. Dodenaeus (1554) mentions many cole crops, but not kohlrabi. In a study by Zeven (1996) where they searched for 16^th^ and 18^th^ century pictures of colecrops, only one painting was revealed that illustrated an intermediate morphology between narrow stem kale and kohlrabi (German painter Jacob Samuel Beck (1715–1778) [19]. This painting however fits with the classification of kohlrabi sister to kale 2 and 3 that consist of several marrow stem kales showing its botanical proximity. The collards form an interesting morphotype, as they are cultivated since the 1800’s in the southern and eastern US, mainly by home gardeners and seed savers [25]. Farnham et al. (2008) mention that their origin is likely from the old world, where they were introduced as early as 1500–1600 by Spanish/Portugese and English settlers [26]. The name collard is likely derived from “colewort”, non-heading kale types. Half of collard accessions are semi-heading, and a hypothesis is that as they were cultivated near cabbages, intermating between collards and cabbage was common. In both our study and the study of Pelc et al. (2015), collards cluster with the cabbages. Chinese kale is an interesting morphotype, as it flowers very early, which is very different from most biannual *B. oleracea*’s that need strong vernalization for flowering. Both summer/autumn and tropical broccoli and cauliflower also have only a weak vernalization requirement. In the SVD-quartets tree, Chinese kale is closest related to the cauliflowers and broccoli clades (AIL), which share the early flowering phenotype due to FLC mutations [18] but are characterized by more mutations (e.g. curd proliferation arrested inflorescence meristems).

Both our study and the study of Mabry et al. (2021) [11] integrated phylogenetic and population genetics techniques with archaeological and historic writing evidence to investigate the evolutionary history of *B. oleracea* crops and its wild relatives. The results in these two studies are largely in agreement with each other. We provided evidence for two domestication lineages (LHL and AIL), which were also present in their individual level phylogeny. We showed that kales are polyphyletic with regard to *B. oleracea* morphotypes, while Mabry et al. also provided evidence for this observation, suggesting highly diverse kale types. Mabry et al. however specifically indicated *B. cretica* as the closest living wild relative of *B. oleracea*, pointing to an Eastern Mediterranean origin. They especially mentioned that five admixture events were identified using TreeMix and highlighted that many admixture events and lineages of exoferal origins characterized the evolutionary history of *B. oleracea*. Among the five admixture events, one is from *B. cretica* accession (198) to a clade of Chinese kale and Tronchuda, a second one from Kohlrabi to another *B. cretica* accession (199), and a third one from Chinese kale to the *B. cretica* accession (199). Interestingly, in our genealogical tree the LHL and AIL formed clear monophyletic groups, while the kohlrabi’s and Chinese kale and Tronchuda’s together were situated at the junction of the AIL and the KA3 clade, which may indicate admixture between these accessions and kale, AIL and possibly wild C9 species like *B. cretica*. Mabry et al. mentioned that *B. cretica* is likely ancestor, however they didn’t find evidence if this is the case for the two main lineages (AIL and LHL). We found evidence for far east origin of the AIL and LHL clades in both the hypothesis of diversified kales and in the first-branching cabbage clade with accessions from the middle-east that seems ancestral. In our study, we propose a scenario in which ancient divergent kale lineages have led to AIL and LHL. This scenario also leads us to support a middle-eastern origin, which corroborates the conclusion of Mabry et al., which is based on identification and inclusion of the closest living relative species, *B. cretica*. Specifically, together with archaeological and literature evidence, we hypothesized that cabbages and cauliflowers stem from kales introduced from Western Europe to the middle-east, possibly transported with the tin-trade routes in the Bronze age, to be re-introduced later into Europe. We estimated the possible timing of the domestication event for *B. oleracea* at ~2560 BC (~4.6 ka). Likewise, the data from Mabry et al. not only supported an origin of cultivation in the Eastern Mediterranean region, but also pointed to a Late-Holocene domestication, a time frame whose boundary (~4.2 ka) is close to our estimation. 

Additionally, we investigated genetic diversity between different groups (i.e. between genebank and modern hybrids) that reflect the history of plant breeding. Overall, allelic variation in genebank accessions was much larger compared to that of modern hybrids, which is important in guiding plant breeders’ strategies. As this study used a very large collection (almost 1000 accessions) we could also compare variation between different morphotypes, different varieties and ecotypes.

In summary, we analysed the genetic diversity, genealogical relationship and population structure among 912 *B. oleracea*- and their wild relative accessions. Our data illustrate that genetic diversity reduces from genebank accessions to modern hybrid accessions. Species tree analysis showed evidence for two lineages, LHL and AIL, with onset of diversification and breeding of cabbages and cauliflowers around 400 BC in the middle east. Different kale and wild *B. oleracea* were likely progenitors of the diverse lineages (besides the AIL and LHL also the sprouts and kohlrabi’s). Cauliflower is the least diverse morphotype and has the strongest genetic differentiation with other morphotypes, which points to a very strong genetic bottleneck.

## Materials and methods

### Plant materials

A set of 912 accessions were collected from both germplasm collections (referred to here as “genebanks”) and modern hybrid cultivars from breeding companies. An accession was defined as one entry in the germplasm/cultivar collection, and represented by a single plant in this study. For details on genebank origin, breeding companies hybrids and “wild C9 species” (non *B. oleracea* species, with the same chromosome number as *B. oleracea*), see Supplementary Notes, Table 1, Table S1–S2.

*B. oleracea* is a self-incompatible species and landraces are thus heterogeneous. To assess this heterogeneity, six accessions, including two cauliflower and four heading cabbage, were selected to study this intra-accession variation. For this purpose, we selected accessions that were either phenotypically uniform or variable when grown in the field. We re-sowed these and randomly genotyped ten individual plants per accession. For cauliflower we included one phenotypically uniform accession (TKI504) and one phenotypically non-uniform accession (TKI506). We also included two uniform heading cabbage accessions (TKI424 and TKI531) and two non-uniform accessions (TKI529 and TKI541) (Table S3). Our analysis indicated that the decision to represent accessions by single plants doesn’t bias the diversity analysis, as generally intra-accession variation is smaller than inter-accession variations, even though the variations are underrepresented by single plants (Supplementary Notes, Fig. S1).

### DNA extraction, sequencing and variant calling

Total genomic DNA was extracted from fresh leaves using an optimized CTAB method [33]. For hybrid accessions (being the result from a cross between two homozygous inbred lines, thus plants from the same accession are genetically identical), we isolated DNA from batches of around 100 seedlings to facilitate DNA isolation, while for genebank accessions, one plant per accession was genotyped; for two cauliflower and four heading cabbage accessions (see Plant materials), DNA was isolated from ten individual plants independently to investigate intra-accession variation. The DNA quality and quantity were measured with Nanodrop 2000. All the DNA samples were genotyped by Sequence-Based Genotyping (SBG) method [34] at Keygene N.V., Wageningen, the Netherlands. The genomic DNA was digested by a combination of PstI (rare cutting) and MseI (frequent cutting) restriction enzymes to reduce genome complexity. After that, primers were annealed and the products were amplified with a 2 basepair (GG) extension to again reduce genome complexity. Constructed SBG libraries were then amplified and sequenced on Illumina Hiseq platform. The generated raw reads were first split for each particular sample according to the 5-bp sequence barcode. No mismatches in the barcode and PstI footprint were allowed. Reads with mismatches in these first 11 nucleotides were left unassigned. After splitting files, barcode and PstI restriction footprint sequences were removed and replaced by the proper PstI restriction sequence. Reads mapping, variant calling and filtering were performed using the method described in Supplementary Notes. An imputed dataset of 14 152 SNPs was utilized for the population genetics analysis.

### Pairwise genetic distance analysis

The R package poppr [35] was used to create a pairwise genetic distance matrix for the 912 accessions with bitwise.dist function. All the genetic distance values were normalized to 0–1 after obtaining the matrix.

### Maximum likelihood (ML) tree construction and population structure analyses

IQ-TREE v1.6.10 [36] was used to construct ML trees for our *B. oleracea* nucleotide version SNP matrix, with the following parameters “-m MFP+ASC -bb 1000 -bnni”. The best model was automatically determined based on the Bayesian Information Criterion (BIC) in the IQ-TREE pipeline, as well as 1000 replicates of ultrafast bootstrapping (UFboot) to estimate node support. The ML tree with bootstrap group frequencies was visualized with interactive Tree of Life (iTOL) (https://itol.embl.de/) [37]. The tree was manually inspected to identify accessions which were clearly mis-named or -identified, clustering in unexpected morphotype groups. Those accessions were not included in the subsequent PCA, STRUCTURE, π and F_ST_ analyses. As reconstructing “phylogenetic” relationships within a species (*B. oleracea*) is actually not possible (only between species), we refer to this tree as a genealogical tree.

Principal component analysis (PCA) was conducted using EIGENSOFT v6.1.4 [38,39] software packages on individual genotypes with default parameters.

Population structure was inferred using STRUCTURE v2.3.4 [29] on the genome-wide SNPs. The *K* value, which was defined as a putative number of ancestral populations, was set from 1 to 15. For each *K* value, STRUCTURE was run 10 times with 10 000 burn-in cycles and 10 000 Markov chain Monte Carlo (MCMC) replicates. The CLUMPAK [40] software was used to estimate optimum number of sub-groups with an ad hoc statistics *Δk* method [41] and to visualize the sub-population membership for each accession.

### Ancestral type reconstruction

To reconstruct ancestral states of the “ecotype” (broccoli, cauliflower) and “variety” (heading cabbage), we recompiled SNP matrices for each morphotype as found in the overall ML analysis and reconstructed a morphotype-specific ML tree using IQ-TREE. Ancestral states were reconstructed treating “ecotype/variety” as a character. Character states we used were: winter, summer/autumn, tropical for cauliflower, winter, summer/autumn for broccoli and red, white, savoy, pointed for cabbages. The character state for Romanesco cauliflower’s was set to “unknown” since it is not clear which ecotype these accessions belong to. Character evolution was reconstructed onto the ML trees using Mesquite v3.61 [42] with the “Trace Character History” option, and using unordered parsimony as criterion.

### Genetic diversity analysis

The average difference per locus over each pair of accessions, π, estimates the level of genomic diversity in a group of accessions [43]. VCF-tools v0.1.15 [44] was used to calculate π for our data with a sliding window size of 100 kb and step size of 10 kb. Reduction of diversity (ROD) metrics was also calculated based on π-value [24, 45]. When calculating ROD, wild *B. oleracea* or genebank group was used as the control group. F_ST_ is the population fixation statistics to calculate the pairwise genomic differentiation between two groups of samples [46]. Pairwise F_ST_ between two different groups was estimated using VCF tools v0.1.15 with a sliding window size of 100 kb and step size of 10 kb. F_ST_ results were interpreted using the same methods described by Del Carpio et al. [47], where the F_ST_ value of 0 denotes no differentiation and 1 denotes complete differentiation between populations. Little differentiation is considered when F_ST_<0.05, moderate differentiation when 0.05 ≤ F_ST_<0.15, strong differentiation when 0.15 ≤ F_ST_<0.25, and very strong differentiation when F_ST_ ≥ 0.25 [48–50].

### Species tree reconstruction

We used SVD-quartets as implemented in PAUP* version 4.0a (build 168) with standard settings, for analyzing a 875 x 14 152 SNP matrix (“Overall”) from which all constant sites had been removed, and in which each terminal had been assigned to one of the 12 morphotypes. Given the overall IQ-TREE genealogy in which Kales and wild *B. oleracea* were on three separate branches respectively, we decided to allow three Kale lineages and three wild *B. oleracea* lineages in the SVDq analysis, each assigned multiple members. After nodes with bootstrap values <50% were collapsed, the resulting overall SVDq morphotype tree topology was then used as a constraint to estimate branch lengths based on the nucleotide version of a subsampled 57 terminal SNP matrix (“SUB”). This matrix, representing all 12 morphotypes, and using wild C9 species as outgroup, was compiled in such a way as to represent (deep) nodes in the Overall ML tree. The “autoModel” option in PAUP* was used to find the best-fitting model and parameter values. Using “saveTrees”, the ML based branch lengths were computed and the tree saved. The next step was to import the tree in Mesquite and use the “arbitrarily make ultrametric” command to produce an ultrametric version of the tree.

## Supplementary Material

Web_Material_uhac033

## Data Availability

The data for this article have been deposited in Figshare (https://doi.org/10.6084/m9.figshare.16338855).
