# Supplementary material for: Evidence for two domestication lineages supporting a middle-eastern origin for Brassica oleracea crops from diversified kale populations
Source: Hortic Res. 2022 Feb 19;9:uhac033. doi: 10.1093/hr/uhac033 (PMC8976692; doi:10.1093/hr/uhac033)
Supplement: Web_Material_uhac033 [file web_material_uhac033.zip › Supplementary information.docx]

**Evidence for two domestication lineages supporting a middle-eastern origin for *Brassica oleracea* crops from diversified kale populations**

**Running title:** A middle-eastern origin for *Brassica oleracea* crops

Chengcheng Cai^1,2^, Johan Bucher^1^, Freek T. Bakker^3^ and Guusje Bonnema^1*^

^1^ Plant Breeding, Wageningen University and Research, Wageningen, The Netherlands

^2^ Graduate School Experimental Plant Sciences, Wageningen University and Research, Wageningen, The Netherlands

^3^ Biosystematics Group, Wageningen University and Research, Wageningen, The Netherlands

**Email address:** Chengcheng Cai, chengcheng.cai@wur.nl; Johan Bucher, johan.bucher@wur.nl; Freek T. Bakker, freek.bakker@wur.nl; Guusje Bonnema, guusje.bonnema@wur.nl

***Correspondence: Guusje Bonnema** ([guusje.bonnema@wur.nl](mailto:guusje.bonnema@wur.nl)), Tel: +31 317484028

**Supplementary Notes**

**Plant materials.** The majority of genebank materials were ordered from the following germplasm collections: Center for Genetic Resources (CGN, the Netherlands), the Germplasm Resources Information Network (GRIN, the USA), Horticulture Research International (HRI) and the Leibniz Institute of Plant Genetics and Crop Plant Research (IPK, Germany), while most of modern hybrid materials were provided by breeding companies of Bejo, Enza, HMClause, Mikadokyowa, Monsanto, Nickerson-Zwaan, Rijk Zwaan, Sakata, Seminis, Syngenta and Takii (Table S1). Besides 879 *B. oleracea* accessions, also three *B. bourgeaui* accessions, one *B. cretica* accession, three *B. drepanensis* accessions, 12 *B. incana* accessions, one *B. insularis* accession, three *B. macrocarpa* accessions, two *B. montana* accessions, four *B. rupestris* accessions and four *B. villosa* accessions were included in this diversity panel (Table 1, Table S2). The *B. oleracea* plant materials consist of the following morphotypes: broccoli, cauliflower, collard green, heading cabbage, Chinese kale, kale, kohlrabi, ornamental kale, Brussels sprouts, tronchuda and wild *B. oleracea*. All of non *B. oleracea* species are wild species with the same chromosome number as *B. oleracea* (2n=2x=18, CC), thus referring as “wild C9 species” in this article.

**Mapping and variant calling.** BWA aln and samse ^1^ were used to map reads from each sample against the reference genome ^2^ with default parameters. SAMtools-0.1.19 package ^3^ was utilized to convert mapping results to individual bam files. Raw variants (referred to here as ‘variant dataset 1’) were called using SAMtools mpileup and BCFtools call ^3^ with parameters “-ug -t DP,DPR,DV,DP4,INFO/DPR,SP” and “-v -m -O v”, respectively. To generate high-quality SNP data (referred to here as “variant dataset 2”) for downstream analysis, BCFtools was further used to perform SNP filtering according to the following criteria: 1) mapping quality, total supporting read depth and SNP quality, each to be ≥30; 2) each SNP to be 3bp away from an InDel; 3) only biallelic SNPs to be retained; 4) for homozygous genotypes, a minimum of 3 reads to support the allele; 5) for heterozygous genotypes, a minimum of 2 reads to support reference and alternative allele respectively; 6) individual genotypes were assigned as missing when the sites failed to pass the above criteria ^4^. For population genetics analysis, the remaining SNPs were filtered to only include those with minor allele frequency (MAF) >2.5% and genotyping rate >80%. We calculated the genotype missing rate for each accession. As the result showed high missing rate (8.3% on average) (Fig. S7), we further performed imputation for missing genotypes using both fillGenotype ^5^ with parameters “-w 20 -k 9 -p -11 -r 0.5” and beagle (Version: 4.1_21) ^6^ with parameters “window=500 overlap=30 niterations=50”. Fillgenotype imputed 1,478,903 missing genotypes and beagle imputed all the missing genotypes. 81.65% missing genotypes were imputed the same by the two methods. To remove imputation false positive, the remaining 19.35% genotypes were still assigned as missing. The imputed variant dataset was named “variant dataset 3”.

After pre-processing for the raw reads (SBG method), we obtained 494.01 Gb clean data in total using Illumina sequencing technology, with an average of 508.24 Mb (4.24 million single-end reads) data for each accession. We then mapped the reads against the *B. oleracea* reference genome ^2^ and called SNPs. We detected 742,169 raw biallelic SNPs (variant dataset1) in total using the mapping results. After strict filtering criteria as described in the section “Materials and methods”, 330,383 high-quality SNPs were obtained (variant dataset 2). We further filtered high-quality SNP dataset (variant dataset 2) according to the criteria of MAF>2.5% and genotyping rate >80%. A total of 14,152 SNP markers (variant dataset 3) were obtained after this filtering. The SNPs were evenly distributed throughout the nine chromosomes of *B. oleracea*, with an overall SNP density of 27.38 SNPs/Mb across the whole genome (Fig. S8, Table S6). As the genotype missing rate for variant dataset 3 was on average 8.3%, we used imputation to reduce the missing rate by 7.07% (Fig. S7). The imputed SNP dataset was utilized for the population genetics analysis of the 912 accessions.

**Intra-accession variation analysis.** Most *B. oleracea* accessions are self-incompatible. As a result, most genebank accessions, representing landraces, are heterogeneous. For SBG genotyping, we extracted DNA of single plants, representing the accession. This clearly results in underrepresentation of allelic variation. We studied this intra-accession variation by genotyping ten individual plants from two cauliflower and four cabbage accessions that varied in phenotypic uniformity when planted in the field. We detected 14,988 SNPs that vary between the ten non-uniform cauliflower plants, four times more than that between the ten uniform cauliflower plants (3,223; Table S3). Similarly, more SNPs were also detected between non-uniform heading cabbage samples compared with that between uniform accession SNPs. Our results reveal that thousands of SNPs vary between intra-accession plants and non-uniform accession harbors more genetic variations than uniform accession. We constructed a maximum likelihood tree including 60 samples representative of the above mentioned two cauliflower and four cabbage accessions, as well as 12 other cauliflower or cabbage accessions, each represented by a single plant. Phylogenetic analysis revealed two distinct clades: one for cabbage and one for cauliflower (Fig. S1). One cabbage sample (TKI529-9) was grouped to cauliflower clade, which is very likely due to sampling mistakes. Within cabbage, both the plants of the uniform and the non-uniform accessions formed distinct clades. Within cauliflower, all plants of the uniform accession (TKI504) formed a clade while plants of the non-uniform accession (TKI506) formed a paraphyletic group (or ‘grade’). Notably, uniform accessions, especially cabbage TKI531 and cauliflower TKI504, have shorter branch length than non-uniform accessions, suggesting that phenotypic uniformity correlates to genetic uniformity. We checked whether accessions represented by single plants clustered separately from the accessions represented by 10 plants each and found that this depended on their distance and the degree of uniformity of the accessions. Our analysis indicated that the decision to represent accessions by single plants doesn’t bias the diversity analysis, as generally intra-accession variation is smaller than inter-accession variations, even though the variations are underrepresented by single plants.

**Pairwise genetics distance.** A few accessions behaved unexpectedly as they differed extensively from their peer accessions. This might be due to incorrect classification of genebank materials. For example, four *B. incana* accessions (WC9_ge_inc12, WC9_ge_inc5, WC9_ge_inc6 and WC9_ge_inc8) were clearly different from the other eight *B. incana* accessions and resembled *B. bourgeaui* accessions since they had similar genetic distance patterns with *B. bourgeaui* accessions. One *B. villosa* accession (WC9_ge_vil2) and one *B. drepanensis* accession (WC9_ge_dre3) are likely *B. incana* (Fig. S2e)*.* This might be due to incorrect classification of genebank materials. *B. bourgeaui* and *B. montana* displayed a large genetic distance to all accessions of other species*.* The single *B. cretica* accession was similar to *B. incana* as they shared a similar genetic distance pattern. Two *B. drepanensis* accessions, all *B. rupestris* accessions and two *B. villosa* accessions also shared similar genetic distance patterns, suggesting that the three species are in close genetic relationship. Similar genetic distance patterns of *B. insularis* and *B. macrocarpa* also indicated that they are genetically close.

**PCA and population structure.** Most of the broccoli modern hybrid accessions were closely clustered in the PCA graphs whereas genebank accessions were not (Fig. S11), again indicating higher level of genetic diversity in genebank accessions. PCA analysis of cauliflower separated the Romanesco types from the rest in the first axis (14.05% explained variation), while winter types separated from the summer/autumn types in PC2 (Fig. S12). However, no strong differentiation between modern hybrid and genebank accessions was observed. For heading cabbage, PC2 evidently separated red cabbage accessions from pointed, savoy and white cabbage accessions (Fig. S13), indicating the latter three cabbage categories shared more similarity in the genetic background than red cabbage accessions. We further conducted PCA analysis for kale and Chinese kale. PC1 clearly set Chinese kale accessions apart from bore and curly kale and marrow stem kale accessions. PC2 evidently separated bore and curly kale accessions from marrow stem kale accessions (Fig. S14). PCA analysis for wild C9 species displayed a cluster mainly containing *B. incana* accessions, separated from another cluster including *B. macrocarpa*, *B. rupestris* and *B. villosa* accessions in PC1 (33.65% explained variation). PC2 (14.17%) set *B. montana* accessions apart from other wild C9 accessions (Fig. S15). This result is consistent with the phylogenetic tree (Fig. S4).Population structure shows that When *K=3*, the majority of heading cabbage accessions formed a new cluster. Ornamental kale and Collard green received more than 50% genetic contributions from heading cabbage. When *K=4*, a new cluster appeared for broccoli with almost half of the accessions highly admixed. Wild C9 species, kohlrabi, Chinese kale, tronchuda, kale as well as wild *B. oleracea* accessions formed a large group, with wild C9 species the least admixed and others receiving more than 50% genetic contributions from wild C9 species. At *K=5*, the large group was divided into three small groups: one group including kohlrabi, Chinese kale and tronchuda, one group including wild C9 species and sprouts, and one group including kale and wild *B. oleracea.* At *K=6*, wild C9 species and sprouts were separated and formed their own independent clusters. At *K=9,* Kohlrabi was separated from Chinese kale and tronchuda*.* At *K=10*, a new cluster appeared for Chinese kale with pure membership and tronchuda received large portion of genetic contributions from Chinese kale. When *K=11*, ornamental kale was separated from collard green.

**Species tree reconstruction.** We used SVDquartets (‘singular value decomposition for quartets’), which is a quartet-based method for generating a species tree ^7^. It is coalescent-based, assuming each site to have its own evolutionary history (given the species tree) and is considered statistically robust under “very general models of molecular evolution” ^8,9^. It is regarded as a statistically consistent estimator of the species tree, even when there is variation in evolutionary rates and effective population sizes ^10^.

The ‘SUB’ matrix, consisting of 57 x 13963 nucleotide SNP’s was executed in PAUP* and the Overall SVDq tree topology enforced. The ‘autoModel’ function in PAUP* indicated GTR+G+I as best-fitting, with the following ‘lset’ and parameter value settings: lset nst=6 rclass=(abcdbe) rmatrix=(0.97292627 3.4077789 1.0291549 0.60527605 3.4077789) basefreq=(0.21799104 0.28239972 0.28154691) rates=gamma shape=1.4995882 pinv=0.072352278 clock=no. Branch lengths were thus calculated and the tree saved in a separate file. Subsequently, after opening the SUB matrix in Mesquite, the tree with branch lengths was imported and visualized, after which it was ultrametricized using Mesquite’s ‘arbitrarily ultrametricize’ command (see Fig. 7).

**BEAST SNAPP.** Using a binary version of the SUB matrix, we applied SNAPP analysis as implemented in the BEAST2 package ^11^, in order to have a separate, independent estimate of both the species tree, its branch lengths and past effective population size *N*_e_ estimates (θ). BEAST SNAPP is a full Bayesian coalescence- based population genetics method computes species tree likelihoods directly from the markers, avoiding having to calculate gene trees for each marker. Using BEAUTi SNAPP we set the following settings: mutation rate U = 1, mutation rate V = 1, coalescence rate = 10, sample; use log likelihood correction; priors: lambda prior was 1/x with initial value = 193.0 and offset = 0; alpha = 11.75, beta = 109.73, kappa = 1, rateprior was gamma distributed, sampled; MCMC: chain length was set at 1M generations, storing a tree every 10k generations. Two separate Markov Chains were run at a Linux workstation and after 146,000 generations the output from each MC was assessed using Tracer ^11,12^. Resulting species trees were summarized using TreeAnnotator and DiversiTree from the BEAST2 package.

For comparison, the SUB matrix (excluding the outgroups C9 species and Wild oleracea’s) was subjected to BEAST SNAPP Markov chain analysis, estimating the species tree in a full Bayesian manner. Unfortunately, the Markov chain was computationally rather demanding causing the set 1M generations to would have finalized in 4 years time. Therefore we interrupted the Markov Chain prematurely at 146,000 generations which took 146 days at a Linux workstation and the resulting log and tree files were processed in Tracer, and Tee-annotator /DensiTree respectively. The SNAPP clade credibility tree (Fig. S10b) is incongruent with both the SVDq tree and overall genealogy topology. Tracer analysis revealed that after 50 days a large increase in LnL was encountered, followed by an additional step-wise increase 20 days later (Fig. S10a), indicating that the Markov Chain had not stabilized yet. ESS values in the SNAPP output were <<200 as measured in Tracer. This lead us to consider the SNAPP Markov chain as not-converged, due to the limited number of generations completed. Nevertheless, we include this result here for comparison as suppl. materials, and in order to use the ancestral effective population size *N*_e_ estimates (θ) (Fig. S10c).

**Supplementary Figures**


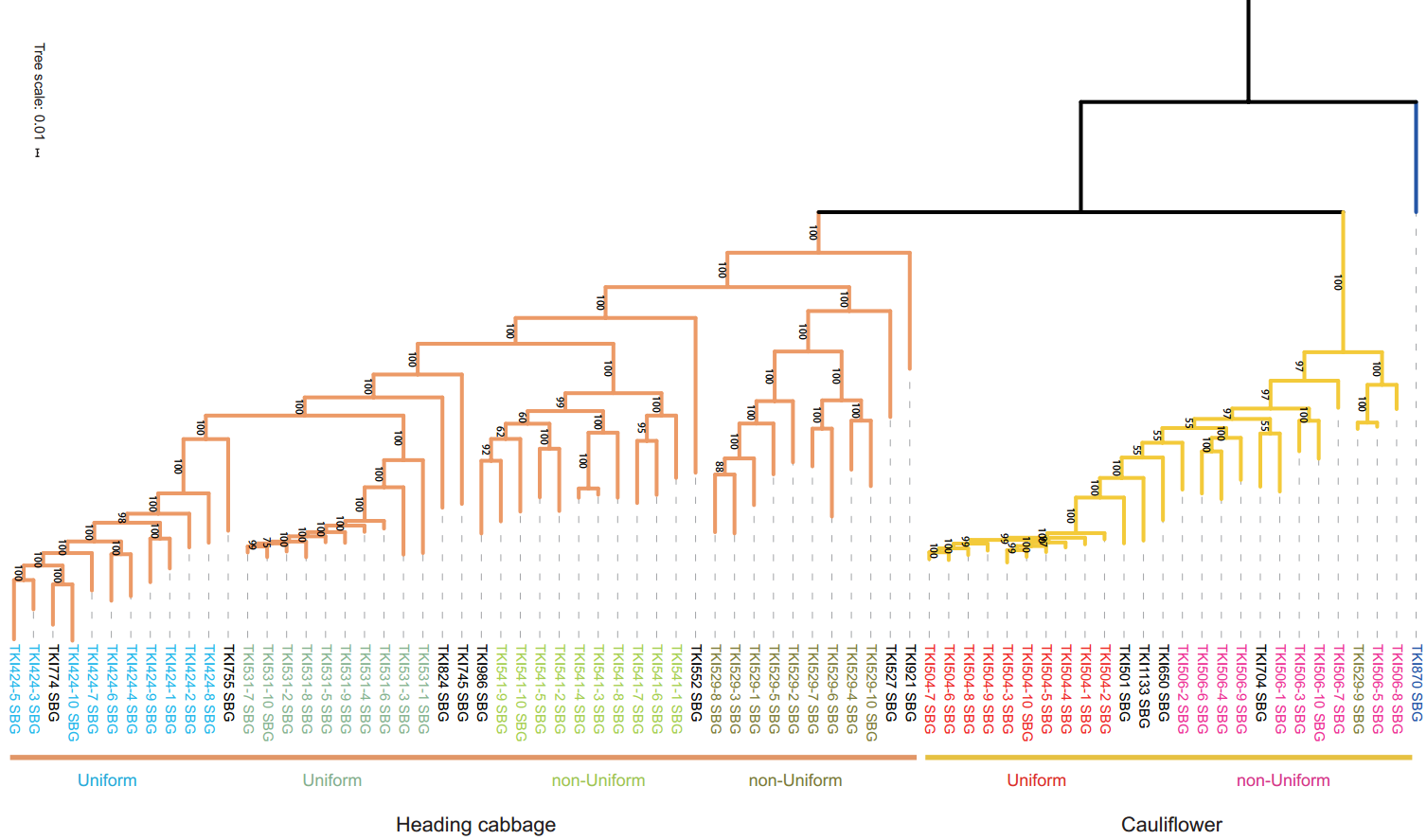


**Fig. S1 Intra-accession variation analysis based on maximum likelihood tree of heading cabbage and cauliflower accessions.** For the selected accessions each (TKI424, TKI531, TKI541, TKI529, TKI504 and TKI506), ten individual plants were genotyped. Other accessions, are represented by one individual plant. TKI870 (*B. montana*, wild C9 species) was used as an outgroup.


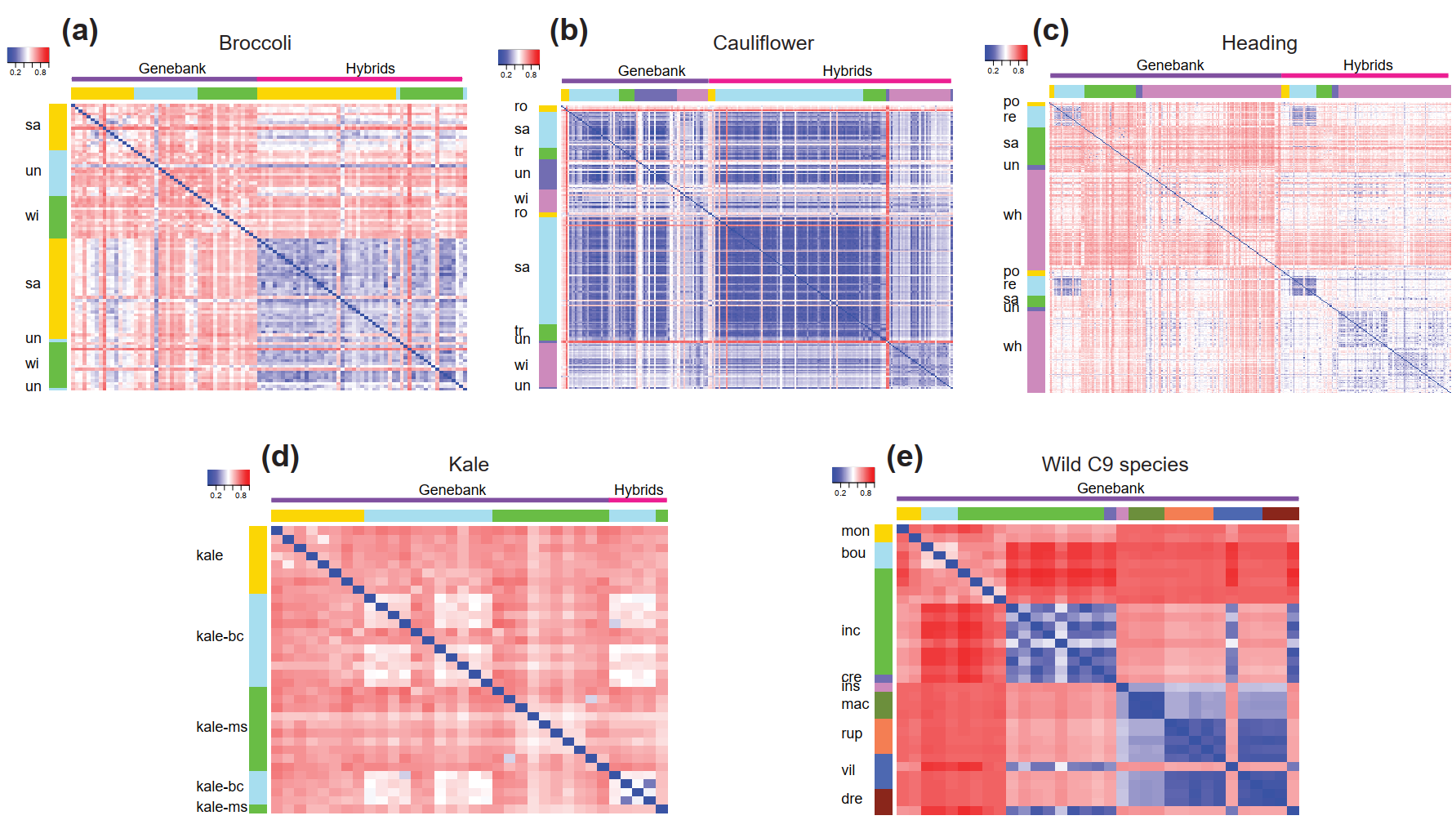


**Fig. S2 Heatmap showing the genetic distance matrix between accessions within given *B. oleracea* morphotypes or within C9 species. Different colors in the vertical bar represent different ecotypes, varieties or species.** **(a)** broccoli (sa: summer/autumn, un: unknown, wi: winter). **(b)** cauliflower (ro: romanesco, sa: summer/autumn, tr: tropical, un: unknown, wi: winter). **(c)** heading cabbage (po: pointed, re: red, sa: savoy, un: unknown, wh: white). **(d)** kale (kale-bc: bore and curly kale, kale-ms: marrow stem kale). **(e)** wild C9 species (bou: *B. bourgeaui*, cre: *B. cretica*, dre: *B. drepanensis*, inc: *B. incana*, ins: *B. insularis*, mac: *B. macrocarpa*, mon: *B. montana*, rup: *B. rupestris*, vil: *B. villosa*).


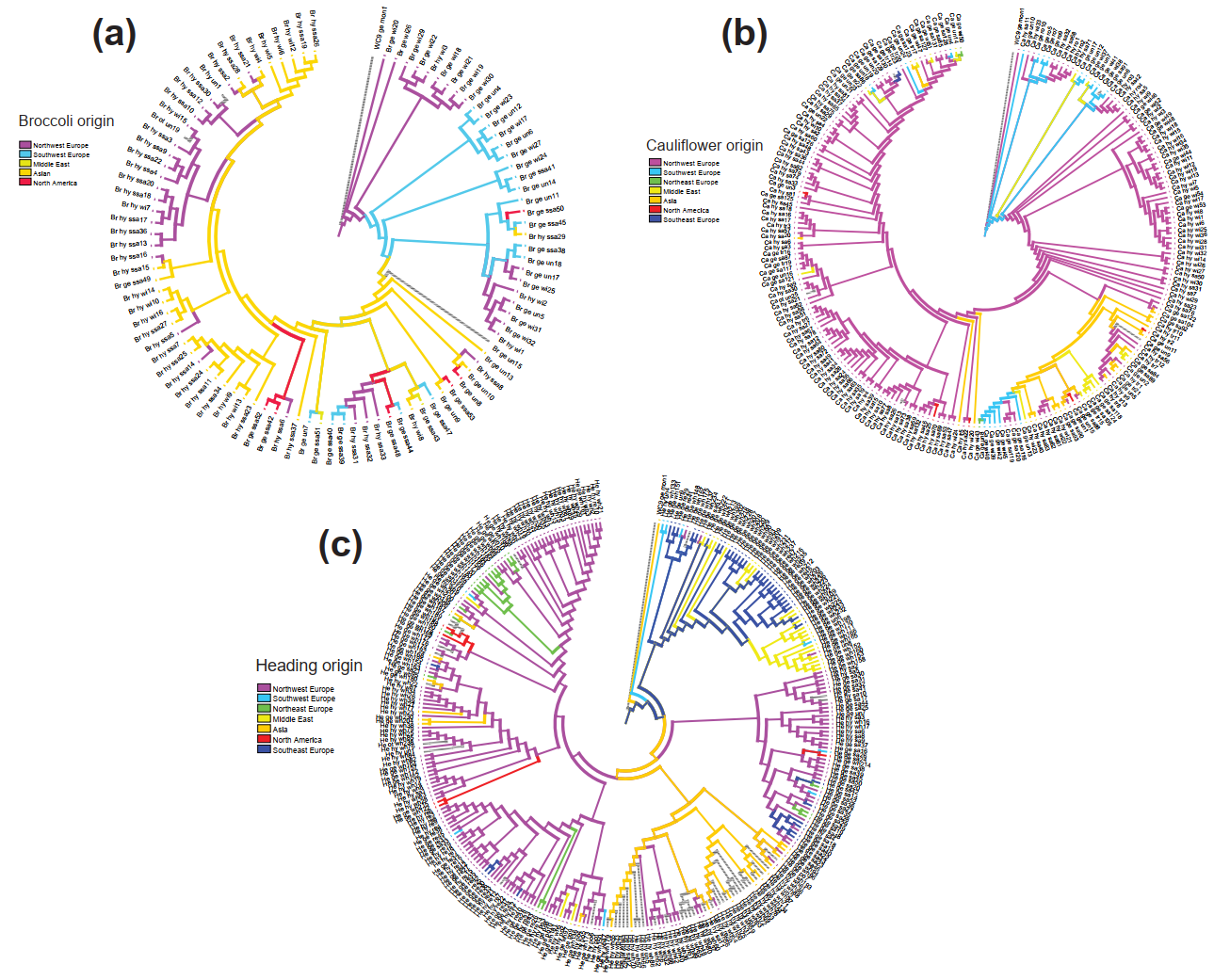


**Fig. S3 Geographical origin character evolution for (a) broccoli, (b) cauliflower and (c) heading cabbage.** Different geographical origins were treated as the states of this character. Grey branches represent accessions with “unknown” state.


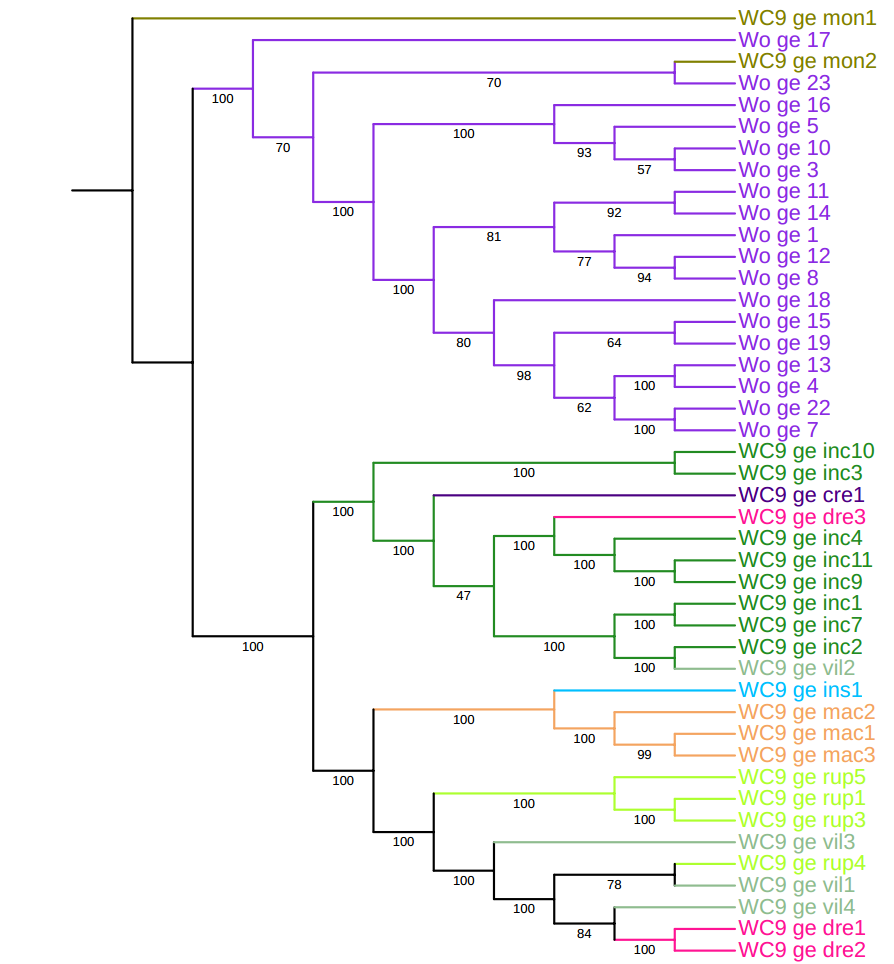


**Fig. S4 Maximum likelihood tree of wild *B. oleracea* and wild C9 species accessions.** The abbreviations: WC9 denotes wild C9 species, Wo denotes wild *B. oleracea*, ge denotes genebank. (bou: *B. bourgeaui*, cre: *B. cretica*, dre: *B. drepanensis*, inc: *B. incana*, ins: *B. insularis*, mac: *B. macrocarpa*, mon: *B. montana*, rup: *B. rupestris*, vil: *B. villosa*).


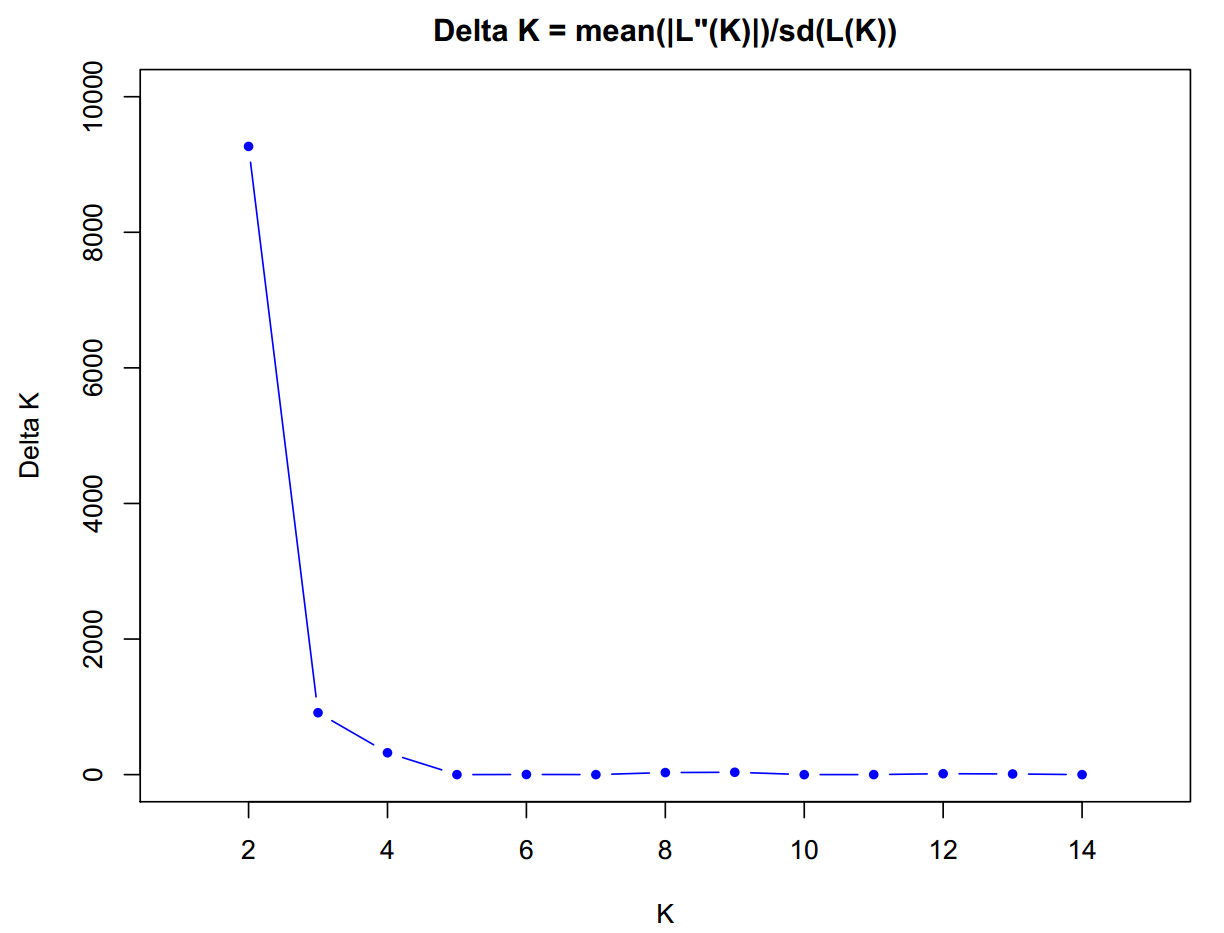


**Fig. S5 *Δk* analysis for the different number of clusters for the *B. oleracea* and wild C9 species accessions.**


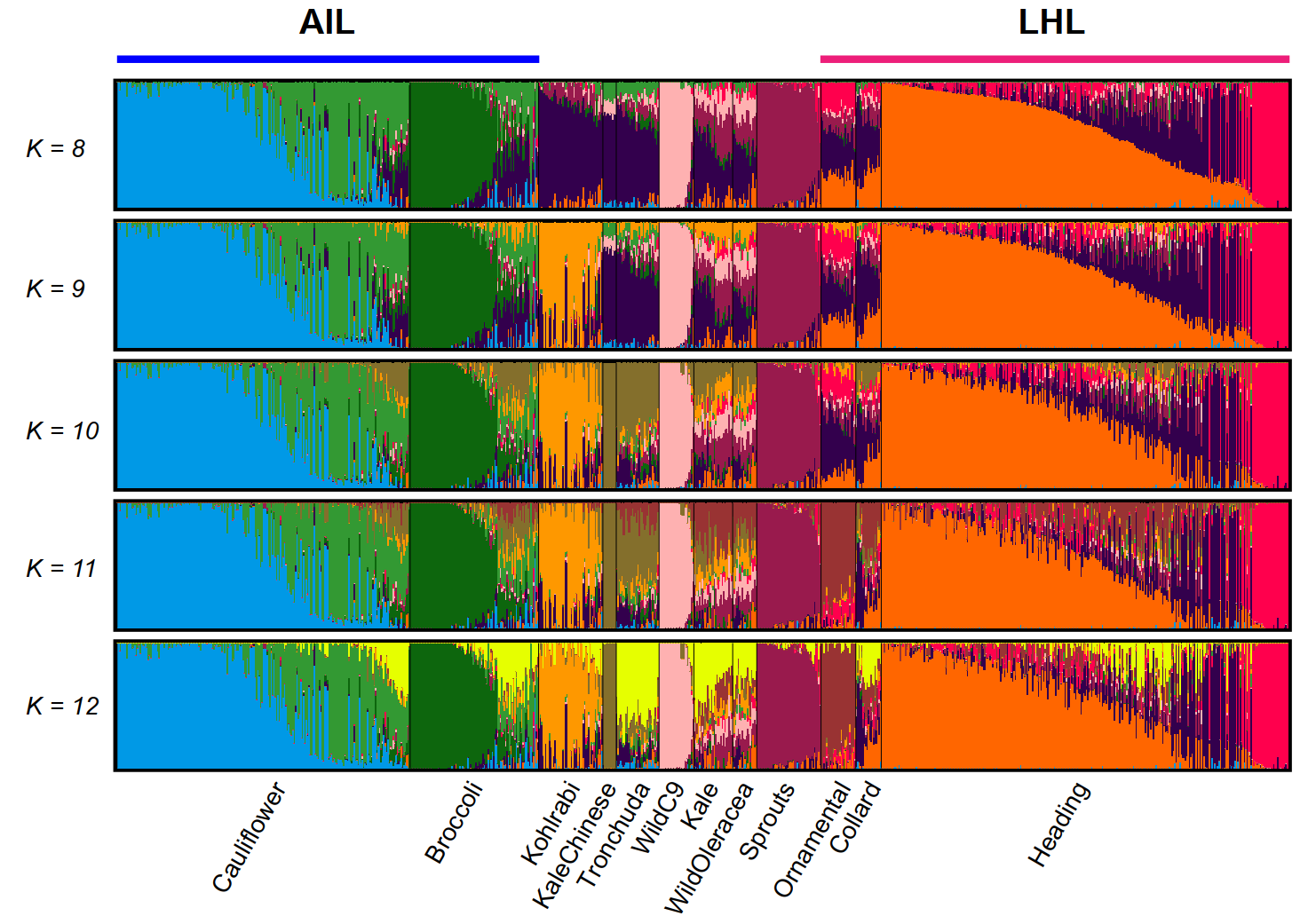


**Fig. S6 Population structure of major *B. oleracea* morphotypes and wild C9 species with different numbers of clusters (*K=8-12*).** Each accession is represented by a vertical bar with different colors. The length of each colored segment in the bar quantifies cluster membership.

**
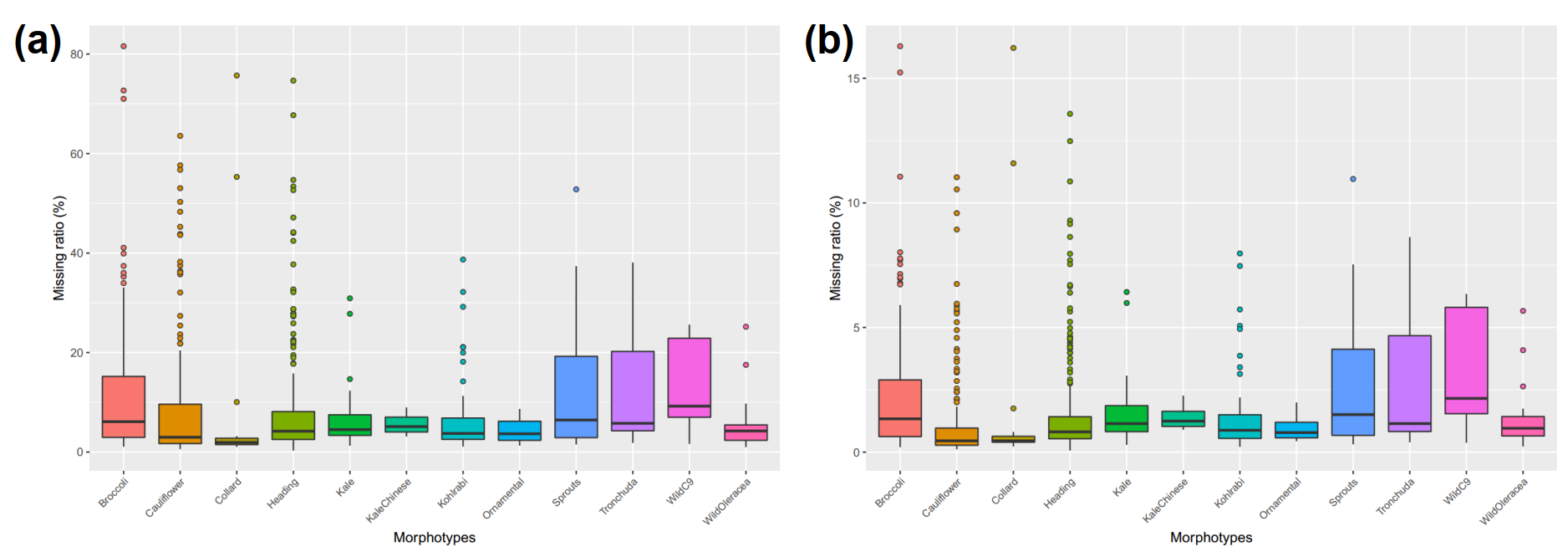
**

**Fig. S7 Boxplot of genotype missing rate for 912 accessions (a) before and (b) after imputation.**


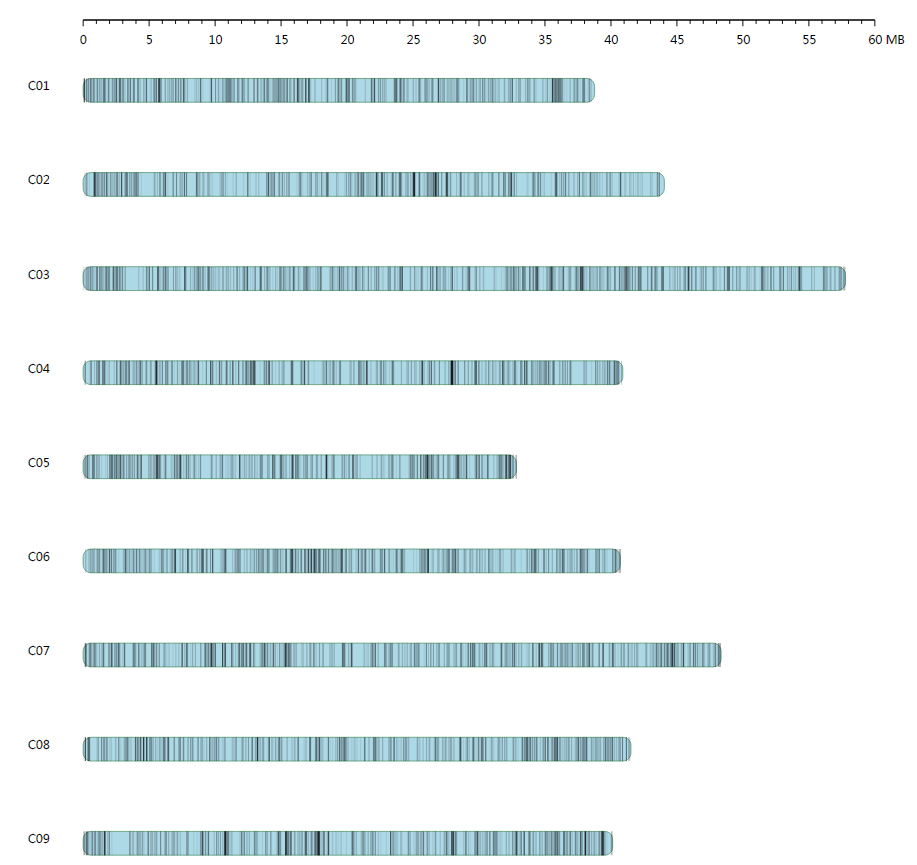


**Fig. S8 Distribution of high-quality SNP markers on the nine chromosomes of *B. oleracea*.**


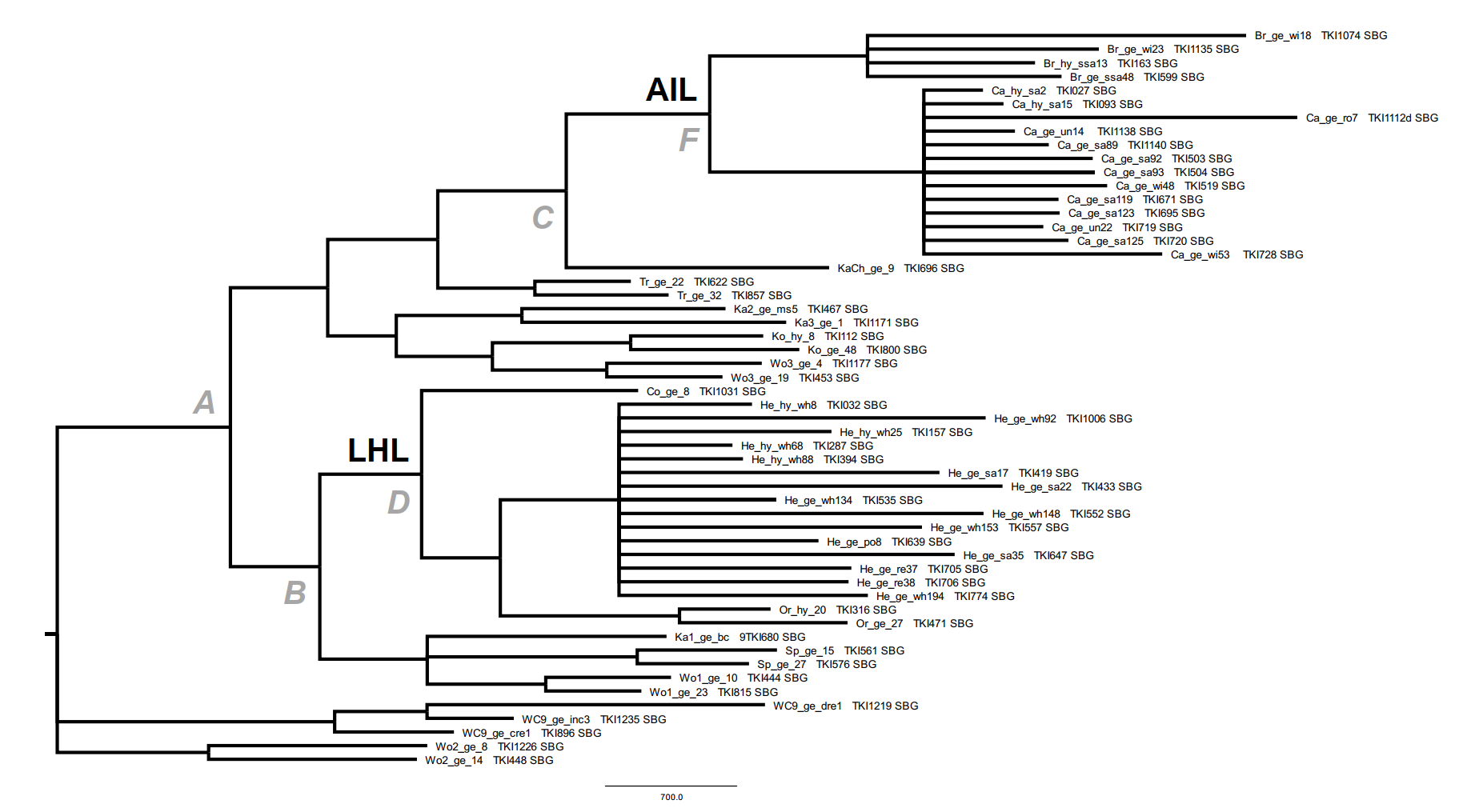


**Fig. S9 Tree topology of the SVDquartet analysis from Figure 7a, with nodes with bootstrap values <50% collapsed, enforced on the SUB matrix (see text).** Branch lengths are according to nucleotide SNP changes.


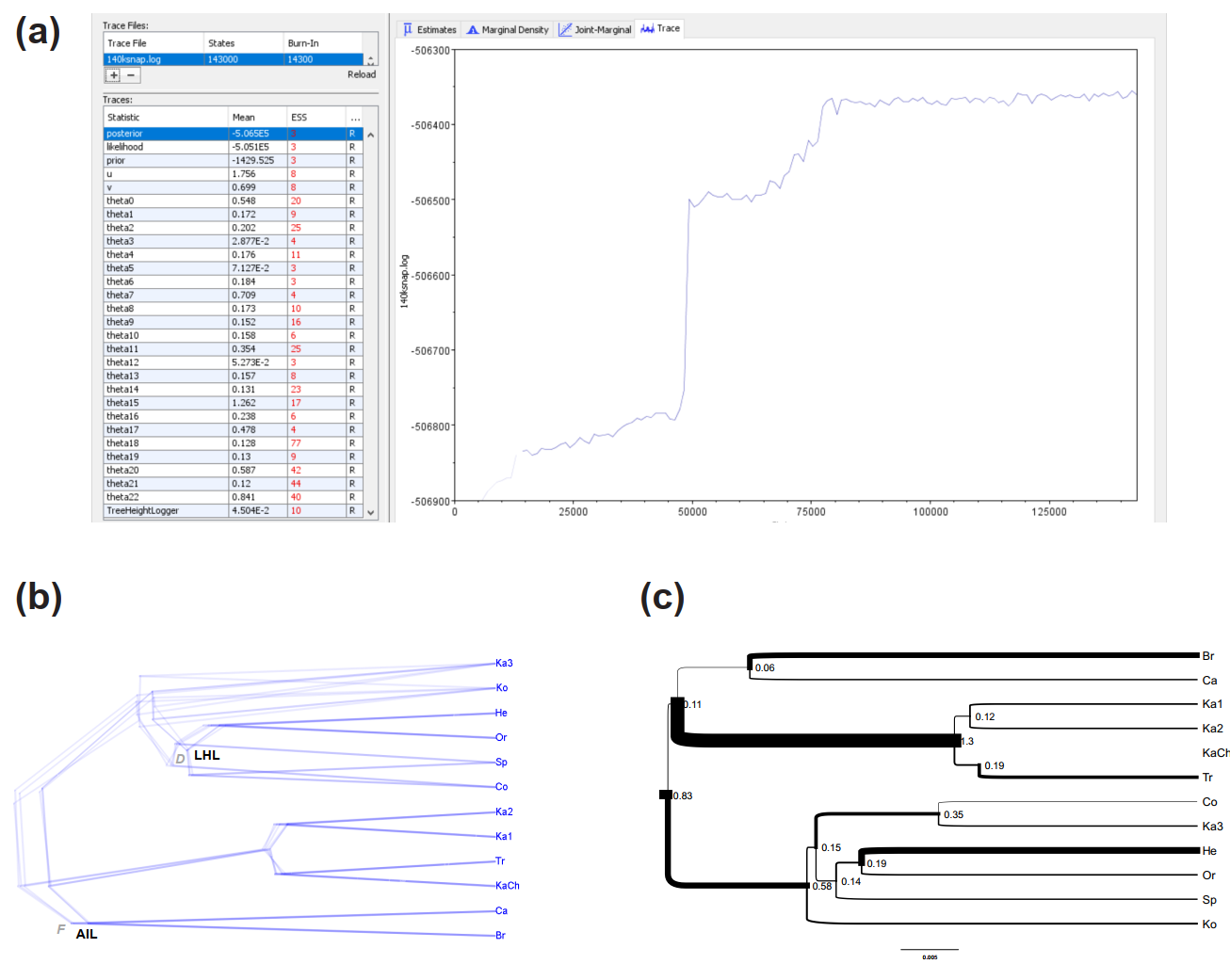


**Fig. S10 BEAST SNAPP analysis.** Preliminary results of a 146k generation Markov Chain with **(a)** Tracer output of the posterior showing the jump-like improvements in posterior after 50k and 80k generations; **(b)** Consensus tree of trees sampled for the last 40k generations; and **(c)** Consensus tree with effective population size *N*_e_ estimates (θ) indicated at the nodes.


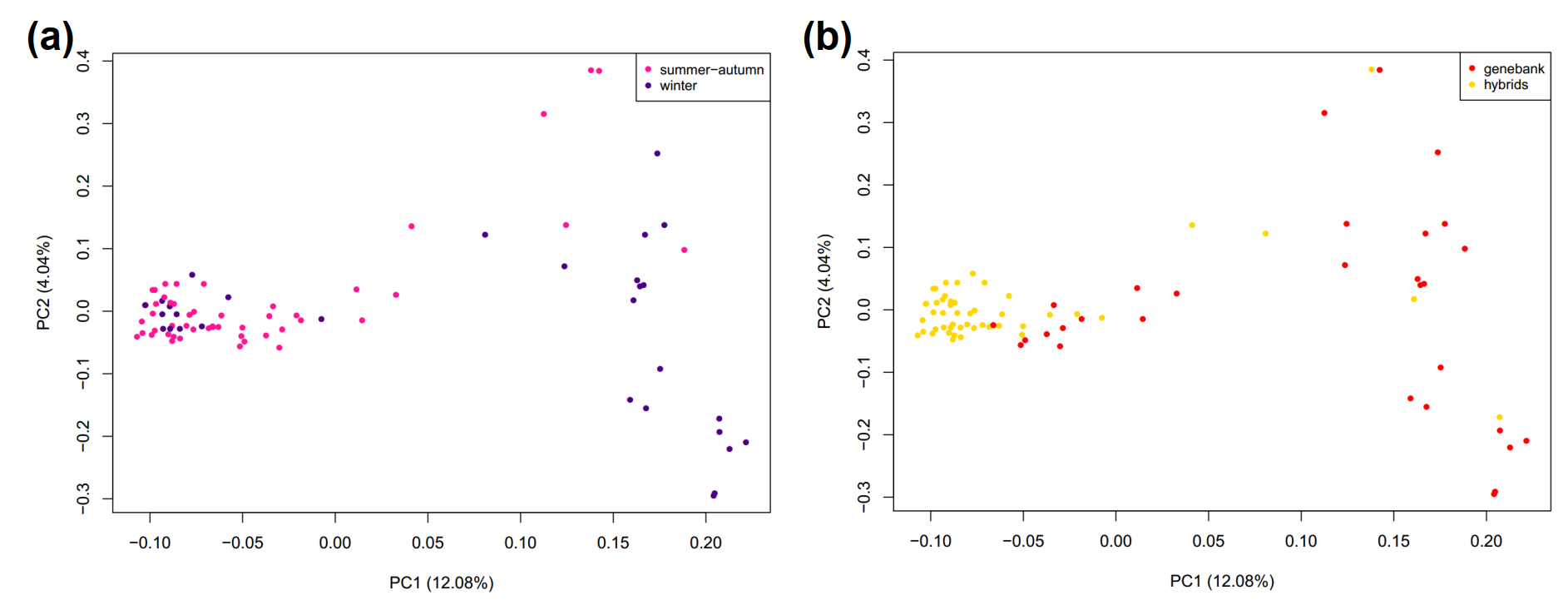


**Fig. S11 PCA plots of broccoli accessions. (a)** Accessions were classified according to different broccoli ecotypes. **(b)** Accessions were classified according to the collection of materials (genebank or modern hybrids). The first two principle components were plotted to visualize the relationships among individuals and groups.


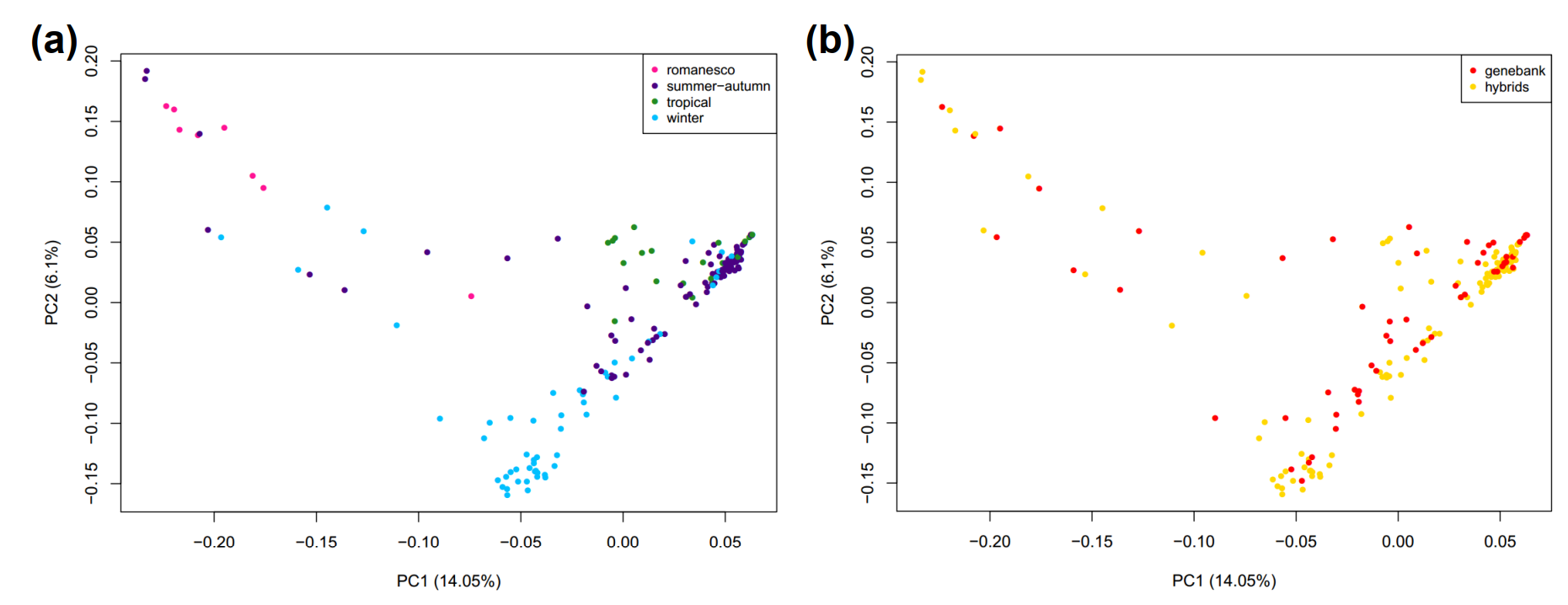


**Fig. S12 PCA plots of cauliflower accessions. (a)** Accessions were classified according to different cauliflower ecotypes. **(b)** Accessions were classified according to the collection of materials (genebank or modern hybrids). The first two principle components were plotted to visualize the relationships among individuals and groups.


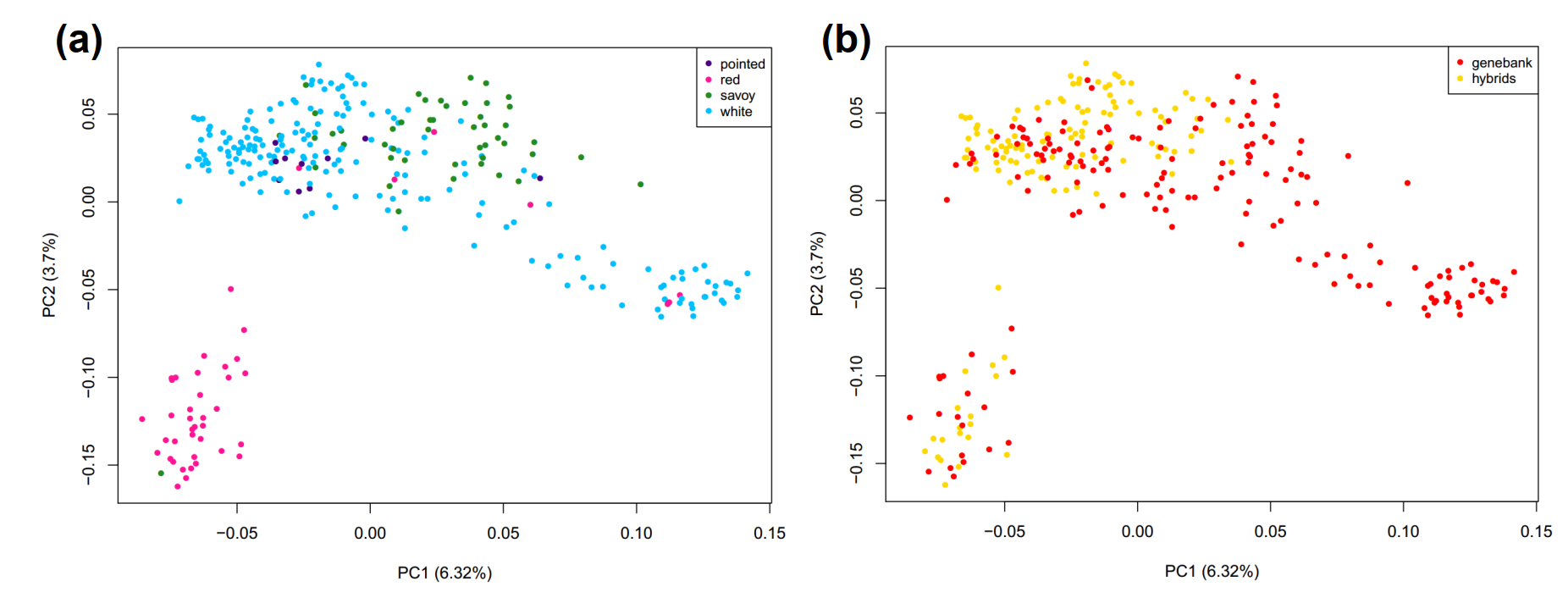


**Fig. S13 PCA plots of heading cabbage accessions. (a)** Accessions were classified according to different varieties. **(b)** Accessions were classified according to the collection of materials (genebank or modern hybrids). The first two principle components were plotted to visualize the relationships among individuals and groups.


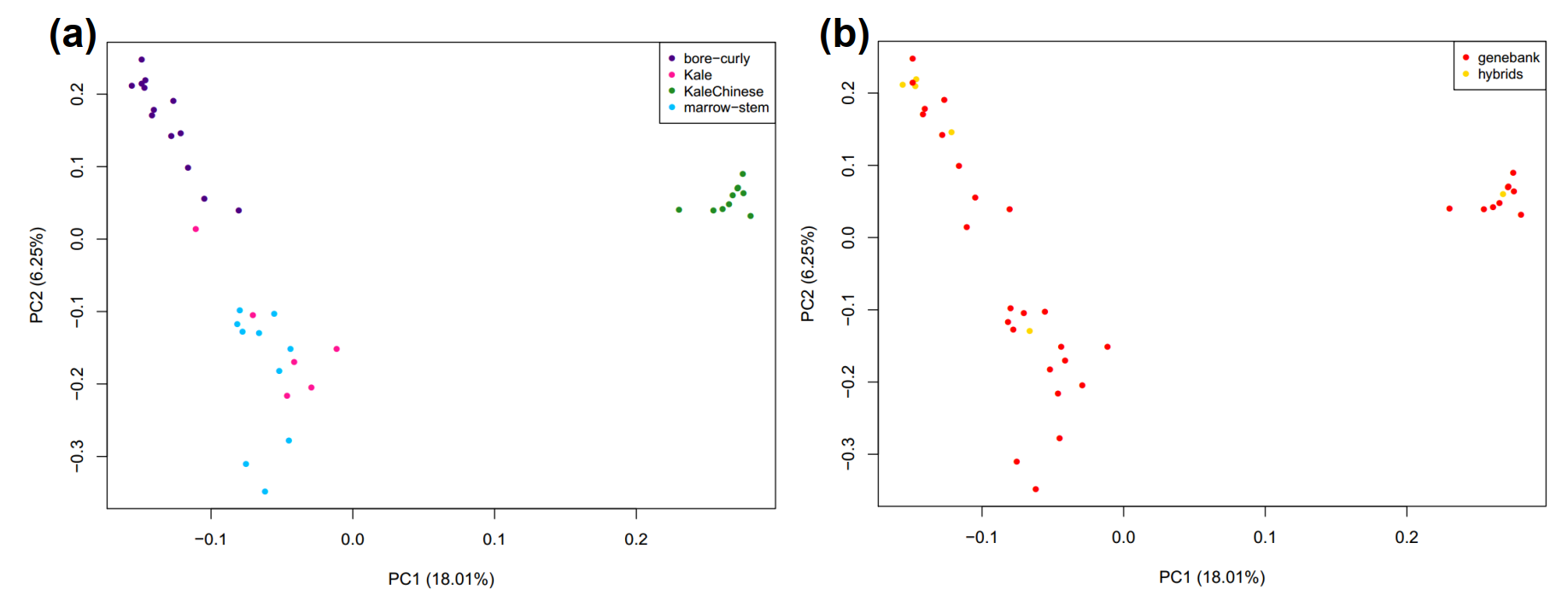


**Fig. S14 PCA plots of kale and Chinese kale accessions. (a)** Accessions were classified according to different kale types. **(b)** Accessions were classified according to the collection of materials (genebank or modern hybrids). The first two principle components were plotted to visualize the relationships among individuals and groups.


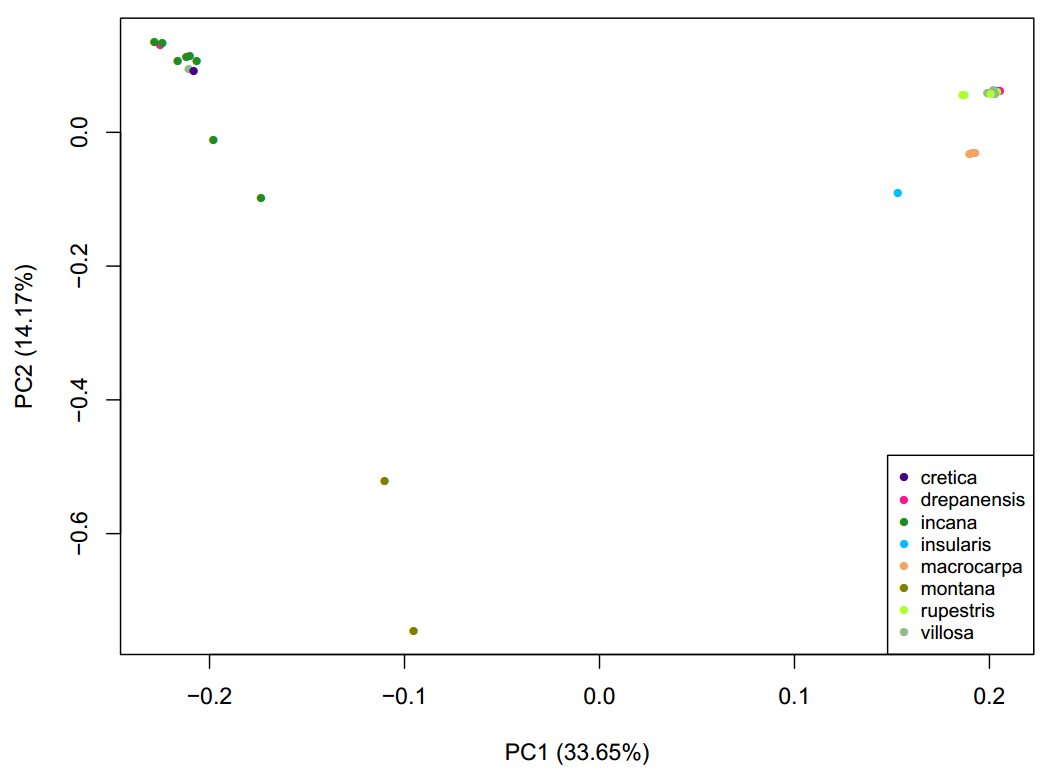


**Fig. S15 PCA plots of wild C9 species accessions.** The first two principle components were plotted to visualize the relationships among individuals and groups.

**Supplementary Tables**

**Table S1 The information of all accessions used in this study. (Excel spreadsheet)**

**Table S2 Summary of wild C9 species accessions.**

| **Species** | **No. Chromosomes** | **No. Samples** | **Total** |
| --- | --- | --- | --- |
| *Brassica bourgeaui* | 9 | 3 | 33 |
| *Brassica cretica* | 9 | 1 |  |
| *Brassica drepanensis* | 9 | 3 |  |
| *Brassica incana* | 9 | 12 |  |
| *Brassica insularis* | 9 | 1 |  |
| *Brassica macrocarpa* | 9 | 3 |  |
| *Brassica montana* | 9 | 2 |  |
| *Brassica rupestris* | 9 | 4 |  |
| *Brassica villosa* | 9 | 4 |  |
| *Brassica hilarionis* | 9 | 0 |  |

**Table S3 The number of SNPs that vary between 10 plants within each accession.**

| **Accession_ID** | **Type** | **No. SNP** |
| --- | --- | --- |
| TKI504 | Cauliflower-Uniform | 3,223 |
| TKI506 | Cauliflower-non Uniform | 14,988 |
| TKI424 | Heading-Uniform | 11,564 |
| TKI531 | Heading-Uniform | 14,763 |
| TKI541 | Heading-non Uniform | 18,666 |
| TKI529 | Heading-non Uniform | 24,888 |

**Table S4 Genome-wide nucleotide diversity (π) and reduction of diversity (ROD) for each group.**

| **Morphotype** | **π** | **No. accession** | **ROD (cul/wildOleracea)** |
| --- | --- | --- | --- |
| Broccoli | 1.03E-05 | 96 | 2.14E-01 |
| Cauliflower | 7.13E-06 | 219 | 4.58E-01 |
| Collard | 1.19E-05 | 19 | 9.39E-02 |
| Heading | 1.04E-05 | 304 | 2.07E-01 |
| KaleChinese | 1.05E-05 | 10 | 2.02E-01 |
| Kale | 1.23E-05 | 29 | 6.21E-02 |
| Kohlrabi | 1.10E-05 | 48 | 1.61E-01 |
| Ornamental | 1.10E-05 | 26 | 1.63E-01 |
| Sprouts | 9.38E-06 | 48 | 2.87E-01 |
| Tronchuda | 1.13E-05 | 32 | 1.44E-01 |
| WildC9 | 9.68E-06 | 26 | 2.64E-01 |
| WildOleracea | 1.32E-05 | 18 | 0.00E+00 |

**Table S5 Pairwise comparison of F_ST_ values between different ecotypes/varieties.**

**Broccoli**

| Group | summer/autumn | winter |
| --- | --- | --- |
| summer/autumn | - | 0.04 |
| winter | - | - |

**Cauliflower**

| Group | summer/autumn | tropical | winter |
| --- | --- | --- | --- |
| summer/autumn | - | 0.02 | 0.06 |
| tropical | - | - | 0.07 |
| winter | - | - | - |

**Heading cabbage**

| Group | pointed | red | savoy | white |
| --- | --- | --- | --- | --- |
| pointed | - | 0.09 | 0.03 | 0.03 |
| red | - | - | 0.09 | 0.06 |
| savoy | - | - | - | 0.04 |
| white | - | - | - | - |

**Table S6 Summary of SNPs on each chromosome of *B. oleracea*.**

| **Chr** | **Chr Len (bp)** | **No. SNP** | **Density (No. SNPs/Mb)** |
| --- | --- | --- | --- |
| C01 | 38,761,720 | 1,144 | 29.51 |
| C02 | 44,046,003 | 1,057 | 24.00 |
| C03 | 57,781,463 | 1,536 | 26.58 |
| C04 | 40,895,475 | 1,135 | 27.75 |
| C05 | 32,828,328 | 992 | 30.22 |
| C06 | 40,704,471 | 1,223 | 30.05 |
| C07 | 48,346,208 | 1,386 | 28.67 |
| C08 | 41,516,064 | 1,140 | 27.46 |
| C09 | 40,126,856 | 1,196 | 29.81 |
| C00 | 131,923,972 | 3,343 | 25.34 |
| Total | 516,930,560 | 14,152 | 27.38 |

**References**

1. Li, H. & Durbin, R. Fast and accurate short read alignment with Burrows–Wheeler transform. *bioinformatics* **25**, 1754-1760 (2009).

2. Liu, S. *et al.* The Brassica oleracea genome reveals the asymmetrical evolution of polyploid genomes. *Nature communications* **5**, 1-11 (2014).

3. Li, H. *et al.* The sequence alignment/map format and SAMtools. *Bioinformatics* **25**, 2078-2079 (2009).

4. Zhang, L. *et al.* RNA sequencing provides insights into the evolution of lettuce and the regulation of flavonoid biosynthesis. *Nature communications* **8**, 1-12 (2017).

5. Huang, X. *et al.* Genome-wide association studies of 14 agronomic traits in rice landraces. *Nature genetics* **42**, 961 (2010).

6. Browning, B.L. & Browning, S.R. Genotype imputation with millions of reference samples. *The American Journal of Human Genetics* **98**, 116-126 (2016).

7. Chifman, J. & Kubatko, L. Quartet inference from SNP data under the coalescent model. *Bioinformatics* **30**, 3317-3324 (2014).

8. Leaché, A.D. & Oaks, J.R. The utility of single nucleotide polymorphism (SNP) data in phylogenetics. *Annual Review of Ecology, Evolution, and Systematics* **48**, 69-84 (2017).

9. Chifman, J. & Kubatko, L. Identifiability of the unrooted species tree topology under the coalescent model with time-reversible substitution processes, site-specific rate variation, and invariable sites. *Journal of theoretical biology* **374**, 35-47 (2015).

10. Long, C. & Kubatko, L. The effect of gene flow on coalescent-based species-tree inference. *Systematic biology* **67**, 770-785 (2018).

11. Bouckaert, R. *et al.* BEAST 2: a software platform for Bayesian evolutionary analysis. *PLoS Comput Biol* **10**, e1003537 (2014).

12. Rambaut, A., Drummond, A.J., Xie, D., Baele, G. & Suchard, M.A. Posterior summarization in Bayesian phylogenetics using Tracer 1.7. *Systematic biology* **67**, 901 (2018).
